# Research on passenger-train coordination and passenger flow evolution in Urban rail transit systems under degraded operations

**DOI:** 10.1371/journal.pone.0349898

**Published:** 2026-07-06

**Authors:** Jing Zuo, Yuanxian Zhao, Junting Lin

**Affiliations:** 1 School of Automation and Electrical Engineering, Lanzhou Jiaotong University, Lanzhou, China; 2 CRSC Research & Design Institute Group Co., Ltd., Beijing, China; Beijing University of Technology, CHINA

## Abstract

Under degraded operations in urban rail transit systems, the coordinated state between passengers and trains undergoes significant changes due to factors such as train frequency reduction. This creates a critical mismatch between passenger demand and transport capacity, leading to rapid passenger accumulation at stations and potential safety hazards from abnormal crowding and systemic congestion. This study develops an integrated simulation model that combines train schedules with passenger arrival patterns to quantify how altered departure sequences generate supply-demand imbalances. A bounded rationality route choice model incorporating four decision-making dimensions – time value, transfer burden, service degradation impact, and route familiarity – captures passenger behavioral adaptations during degraded operations. The experimental results indicate that under abnormal train operation scenarios, the model incorporating bounded rationality assumptions is closer to actual observed values compared with the fully rational benchmark model. In the L2 degradation scenario, the deviation between the bounded rationality model and the actual values is controlled within 0.6%–1.3%, whereas the deviation of the fully rational model is approximately 12%. Concurrently, passenger flow propagation intensity in core sections rises from 0.3 to 0.7, showing a clear evolution from localized accumulation to regional diffusion. The research provides an effective methodology for identifying passenger flow dynamics under degraded operational scenarios and supports decision-making for service optimization and passenger flow management.

## 1. Introduction

Urban rail transit network operations have led to sustained growth in passenger flow volumes, particularly in major metropolitan areas where peak-hour demand demonstrates high spatiotemporal concentration and significant volume-capacity imbalances [1]. When service degradation necessitates a transition from Communication-Based Train Control (CBTC) to intermittent or interlocking control modes, the reduced signaling capacity triggers substantial operational adjustments including decreased train frequencies and modified turnaround schemes. This capacity-demand mismatch results in platform overcrowding, station congestion, and train overloading conditions. Current passenger flow assessment during degraded operations primarily depends on station staff observations and automated fare collection system data, approaches that only support subjective evaluations and discrete measurements while failing to accurately capture evolving spatiotemporal passenger distribution patterns under emergency service plans [2,3]. Furthermore, passenger travel behavior exhibits complex, dynamically-coupled characteristics where route choices continuously adapt to waiting times and crowding conditions through multi-factor interactions. These operational challenges collectively hinder the direct observation and precise estimation of passenger flow dynamics during service disruptions. Therefore, research on human–train collaborative passenger flow estimation is critically important. Such research contributes to understanding evolving crowd movement patterns and developing effective emergency response strategies aligned with actual passenger behavior characteristics.

Existing research on urban rail transit passenger flow primarily concentrates on conventional passenger forecasting [4–6] and network-wide volume prediction under large passenger flow scenarios and major events [7–10]. These investigations utilize multi-source data including historical AFC records, meteorological conditions, and temporal factors, adopting data-driven methodologies with various intelligent learning approaches to achieve precise macro-level passenger flow trend predictions. However, such macro-static analyses prove inadequate for intuitively characterizing the dynamic propagation processes and evolutionary trends of passenger flow distribution across network systems. Consequently, researchers have extensively employed simulation and dynamic modeling techniques to examine urban rail transit network characteristics and passenger flow propagation mechanisms. At the network level, Huang et al. [11] established a weighted coupled map lattice model to construct a P-space passenger flow network, comprehensively evaluating metro network dynamic stability across multiple dimensions including connectivity, adaptability, and effectiveness while revealing its evolutionary patterns alongside network structural changes. Li et al. [12] enhanced the coupled map lattice model to address the characteristic high station density in large-scale rail transit networks, specifically analyzing cascade failure interaction mechanisms between adjacent and coupled stations, with findings indicating that passenger flow’s impact on cascade failures approximates nine times that of topological structure. At the microscopic level, several scholars have introduced cellular automata methodology to develop station crowd evacuation dynamics models [13], station congestion diffusion evolution models [14], and large passenger flow congestion propagation simulation models [15], enabling detailed characterization of passenger localized interactions and movement patterns within spatially constrained environments. Comparatively, Susceptible-Infected-Recovered (SIR) and Susceptible-Infected (SI) models, featuring minimal parameters, computational efficiency, and intuitive representation of congestion propagation rates, have been widely adopted to characterize passenger flow diffusion processes. For instance, Xiong et al. [16] implemented the SIR model to quantify congestion propagation rates under sudden passenger flow surges, revealing rate fluctuation patterns during peak hours and at transfer stations. Jia et al. [17] applied an improved SIR epidemic model to quantify congestion propagation throughout multi-level rail transit networks, elucidating how propagation rate, node degree, and recovery rate collectively influence congestion scope. Shi et al. [18] introduced the epidemiological SIR model into the study of urban rail transit congestion, constructing a dynamic propagation model to reveal the transmission mechanism and comparing the effectiveness of supply-side and demand-side control strategies. Although these studies have illuminated congestion phenomena under both sudden passenger flow conditions and operational disruptions, they mainly focus on propagation rate, impact scope, and chain reactions. However, they have not effectively incorporated microscopic passenger behavior analysis. In practical terms, passenger route reselection behavior has been empirically identified as a crucial microscopic mechanism driving congestion propagation. Certain researchers have introduced behavioral economic frameworks including random regret minimization [19], cumulative prospect theory [20], and mental accounting [21] to develop enhanced passenger behavior models, thereby strengthening explanatory capacity regarding passenger route choice behavior in uncertain environments. Nevertheless, these methodologies remain insufficient for comprehensively capturing how multi-attribute factors interactively influence travel behavior, while also lacking effective characterization of real-time constraint effects between individual passenger decision-making processes and operational environmental conditions.

To address these research gaps, this paper makes three innovative contributions:

(1) A human-train collaborative simulation framework is constructed, establishing bidirectional feedback mechanisms between trains and passengers. This framework enables dynamic simulation of train operation states and passenger flow behaviors under both normal and degraded operating conditions. It thereby resolves the limitation of existing studies in adequately revealing the endogenous characteristics of passenger flow congestion propagation during service degradation scenarios.
(2) A bounded rationality route reselection model incorporating service degradation effects is developed. By integrating four dimensions—time value, transfer burden, degradation impact, and route familiarity—within a Logit model framework, the model characterizes passenger route choice behavior under degraded operations. The incorporation of degradation effects enables more accurate representation of how service reliability decay influences passenger decision-making processes.
(3) A multi-scale hybrid modeling approach combining propagation models with agent-based simulation is proposed. This approach achieves cross-scale analysis linking macroscopic propagation patterns with microscopic behavioral interactions, establishing an integrated analytical method that effectively bridges macro-level spatiotemporal characteristics of passenger flow with micro-scale passenger behavior.

## 2. Human-train coordinated passenger flow propagation in real train operations

**Fig 1:**
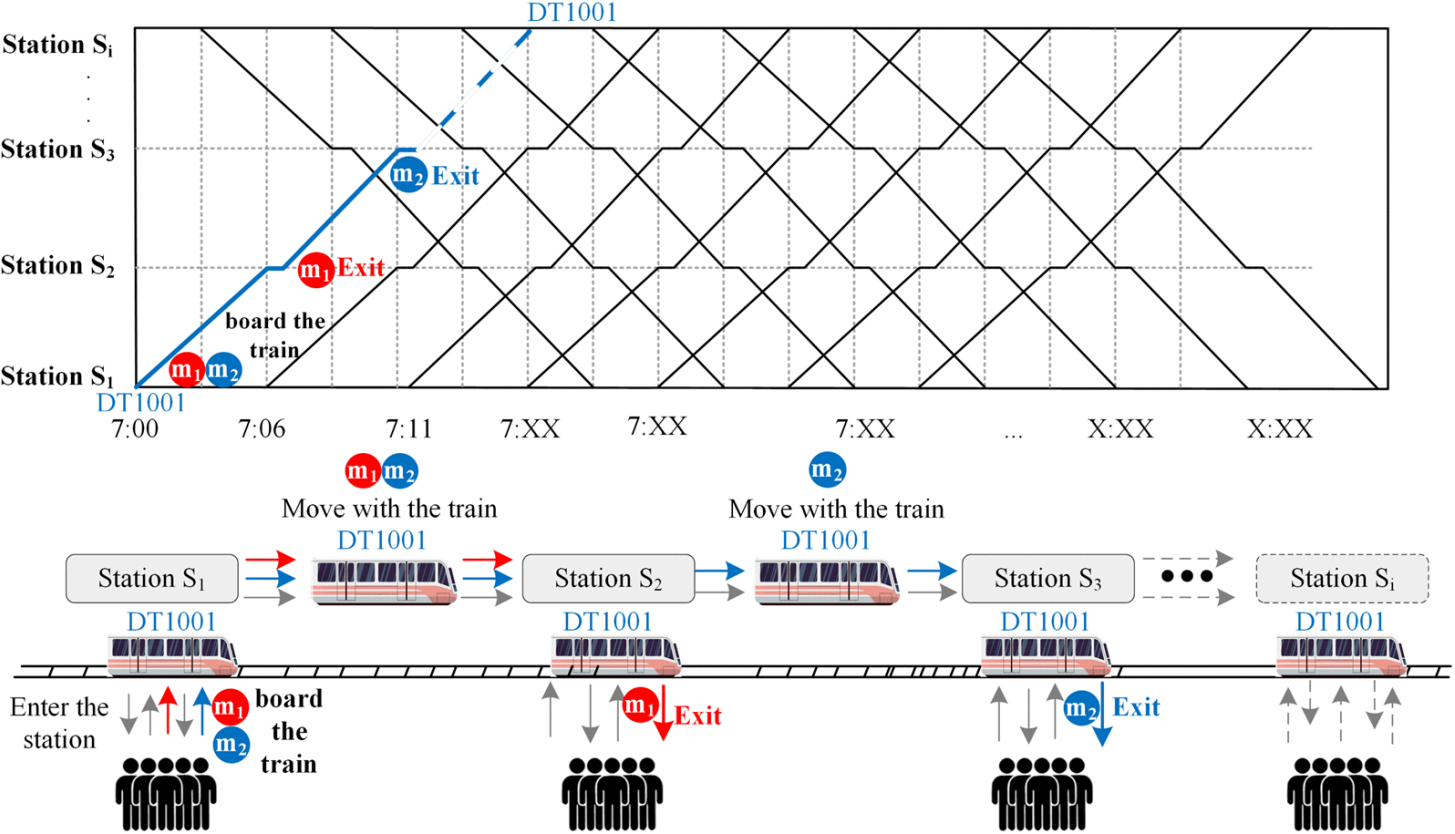
Schematic diagram of the metro passenger flow propagation process.

The human-train coordinated passenger flow propagation process is defined as a propagation process in which the train serves as the carrying medium, and passenger travel is dynamically coupled with the actual operation of the urban rail system. As shown in Fig 1,train DT1001 arrives at station *S*_1_ at 7:00 according to the timetable. Passengers *m*_1_ and *m*_2_ board the train at station *S*_1_ and move with the train. When the train arrives at station *S*_2_,passenger *m*_1_ reaches the destination and alights,i.e.,*m*_1_:*S*_1_ → *S*_2_.Passenger *m*_2_ continues to travel with the train to station *S*_3_, and the propagation process of *m*_2_ is represented as: *S*_1_ → *S*_2_ → *S*_3_.

**Fig 2:**
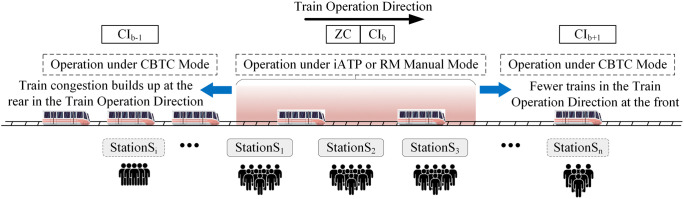
Spatiotemporal distribution characteristics of train operations and passenger flow under degraded conditions.

As illustrated in Fig 2, for any interlocking section CI_*b*_, the preceding and succeeding sections are denoted as CI_*b*+1_ and CI_*b*-1_, respectively, where *b* represents the index of the section and *b*∈{1,2,…,*B*}. When train operation within interlocking section CI_*b*_ is downgraded from the CBTC control mode to the fixed-block ATP mode or RM mode due to signal equipment failures or other causes, the minimum train headway increases, thereby reducing the capacity of the interlocking section.

Following the degradation of the operation mode in interlocking section CI_*b*_, the train headway within the section further increases, resulting in a reduction in the passenger-carrying capacity of the line, extended passenger waiting times, and aggravated passenger accumulation. Train operation in the preceding section CI_*b*-1_ is obstructed, while the train supply capacity in the succeeding section CI_*b*+1_ becomes insufficient [22]. Collectively, these conditions reduce the efficiency of passenger flow evacuation within stations and give rise to a bidirectional coupling imbalance between train operation and passenger flow distribution. Keep the following in mind. There must always be at least two sentences between headings of different levels.

## 3. Dynamic path identification model for Urban rail transit passengers based on bounded rationality in travel decision-making

To accurately characterize passengers’ route decision-making behavior under degraded operations, this chapter sequentially develops a dynamic route identification model and a bounded rationality route choice model, aiming to reveal the intrinsic behavioral mechanisms of passenger flow propagation.

### 3.1. Ethics statement

This study is a transportation system modeling and simulation study based primarily on secondary analysis of publicly available operational data.In addition, questionnaire surveys were conducted to obtain parameters related to passenger behavioral characteristics, namely waiting time tolerance. All participants were informed prior to participation that the survey was conducted solely for academic research purposes, and that the data would not involve any personally identifiable information or commercial use. Participation was voluntary, and respondents indicated their consent by selecting an agreement option before completing the questionnaire. No minors were involved in this study.3.2 Design of Path Generation and Trajectory Identification Algorithms

The urban rail transit network is modeled as a directed graph G=(S,E), where $S=\left\{S_{i}|i=1,2,\text{\{\textbackslash{}ldots}\},n\right\}$ denotes the set of stations and $E=\left\{e_{ij}|i\neq j\right\}$ denotes the set of directed edges from station $S_{i}$ to station $S_{j}$. For route search, an improved Depth-First Search (DFS) algorithm is used to construct a candidate path set $P=\left\{P_{q}|q=1,2,\text{\{\textbackslash{}ldots}\},Q\right\}$, where each path $P_{q}$ connects the entry station $S_{o}$ with the exit station $S_{d}$. A cost-driven mechanism is introduced into DFS, forming a Cost-Driven Depth-First Search (CDFS) algorithm. This approach evaluates paths in real time and eliminates inefficient ones through a cost function:


$$\begin{array}{c} W(P_{q})=\sum_{e_{ij}\in E:e_{ij}\subset P_{q}}t_{e_{ij}}+\alpha \cdot \overset{walk}{\underset{P_{q}}{t}}+\beta \cdot \overset{trans}{\underset{P_{q}}{\delta }} \end{array}$$ (1)

where $t_{e_{ij}}$ denotes the running time of link $e_{ij}$, and $\sum_{e_{ij}\in E:e_{ij}\subset P_{q}}t_{e_{ij}}$ represents the summation of running times over the links that constitute path $P_{q}$, $\overset{\text{walk}}{\underset{P_{q}}{t}}$ is the walking time during transfers, and $\overset{\text{trans}}{\underset{P_{q}}{\delta }}$ is the transfer indicator, equal to 1 if a transfer occurs and 0 otherwise. The parameter α reflects walking-time sensitivity, with larger values indicating stronger sensitivity, while β reflects transfer penalty intensity, with larger values imposing stronger penalties on transfers.

Since depth-first search may generate routes with significantly higher costs during path expansion, to prevent such routes from entering subsequent analysis and to control the search space, the minimum route cost is introduced as a reference to record the smallest generalized cost among the currently generated routes [23]. At the initial stage of the search, $W_{\min}$ is not assigned a value. Once the first complete route $P_{q}$ is generated, its cost is assigned to $W_{\min}$. During subsequent route generation, $W_{\min}$ is updated according to the following rule:


$$\begin{array}{c} W_{\min}=\min(W_{\min},W(P_{q})) \end{array}$$ (2)

Based on the current $W_{\min}$, if the cost of a route $P_{q}$ satisfies $W(P_{q})>2W_{\min}$, the further expansion of this route is terminated. The final retained route set is expressed as:


$$\begin{array}{c} P=\{P_{q}\in P|W(P_{q})\leq 2\cdot W_{\min}\} \end{array}$$ (3)

After the generation of candidate routes is completed, route identification is performed within the constructed origin–destination (OD) candidate route set to determine the path that matches the passenger’s actual travel process. Automated Fare Collection (AFC) data provide information on passengers’ entry station, exit station, and corresponding timestamps, which serve as observed inputs for route identification. Accordingly, the travel attribute vector of passenger *m* is defined as:


$$\begin{array}{c} {𝐀}_{m}=(\overset{m}{\underset{o}{S}},\overset{m}{\underset{d}{S}},\overset{m}{\underset{o}{t}},\overset{m}{\underset{d}{t}}) \end{array}$$ (4)

where $\overset{m}{\underset{o}{S}}$ and $\overset{m}{\underset{d}{S}}$ denote the entry and exit stations of passenger mmm, respectively, and $\overset{m}{\underset{o}{t}}$ and $\overset{m}{\underset{d}{t}}$ represent the corresponding entry and exit times. Equation (4) reflects only the passenger’s observed travel information and serves as the input for route identification, without involving route generation or evaluation. Within the generated OD candidate route set, the observed travel time information provided by Eq. (4) is used to perform temporal matching with candidate routes.

Within the generated OD candidate route set, the observed travel time information provided by Eq. (4) is used to perform temporal matching with candidate routes.The route identification is based on Eq. (4), which calculates the theoretical travel time $t_{P_{q}}$ for the passenger route $P_{q}$ and compares it with the actual observed travel time of the passenger $t_{obs}=\overset{out}{\underset{S_{o}}{t}}-\overset{in}{\underset{S_{d}}{t}}$. The time consistency condition is satisfied if:


$$\begin{array}{c} |t_{P_{\text{q}}}-t_{\text{obs}}|\leq \varepsilon \end{array}$$ (5)

If satisfied, the route is valid. Typically, ε is determined according to the train’s stopping time. For transfer routes, the interval between the current train’s arrival and the transfer train’s departure must meet the minimum transfer time requirement:


$$\begin{array}{c} \overset{dep}{\underset{trans}{t}}-\overset{arr}{\underset{now}{t}}\geq \mathrm{\backslash Updelta}t_{\min} \end{array}$$ (6)

where $\overset{arr}{\underset{now}{t}}$ and $\overset{dep}{\underset{trans}{t}}$ denote the arrival time of the current train and the departure time of the transfer train, respectively, and $\mathrm{\backslash Updelta}t_{\min}$ represents the minimum transfer time, which depends on station scale, transfer complexity, and passenger demand, and usually requires dynamic adjustment.

Under the constraints of time consistency and feasibility, the identified path for passenger m is denoted as $\overset{\left(m\right)}{\underset{q}{P}}$, where m ranges from 1 to M. This path represents the most realistic option within the candidate set and serves as the final identification result.

### 3.2. Passenger behavior evolution and human–train cooperative simulation modeling

**Fig 3:**
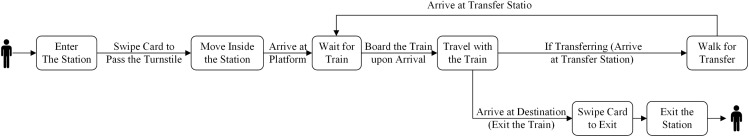
Passenger state transition diagram for urban rail transit.

As shown in Fig 3, passengers sequentially experience entering the station, walking, waiting, boarding, in-train movement, transferring, and exiting. These stages characterize the dynamic evolution of the passenger–train interaction process.

Fig 3 illustrates the dynamic migration simulation of passenger travel through coordination with train operations. During the simulation, passenger walking time is typically assumed to follow a uniform distribution. Once the walking process is completed at time $\overset{m}{\underset{plat}{t}}$, the passenger enters the waiting or transfer state. To realize passenger–train coordination, the train state must be satisfied, and the state judgment function φ(t) is defined as follows:


$$\begin{array}{c} \varphi (\overset{m}{\underset{plat}{t}})=\left\{ \begin{array}{cc} 1, & \overset{dep}{\underset{S_{i}}{t}}\leq \overset{m}{\underset{plat}{t}}\leq \overset{arr}{\underset{S_{i}}{t}}\;\cap \;C_{v}(\overset{m}{\underset{plat}{t}})<0.9\cdot \overset{max}{\underset{v}{C}}\\ 0, & \text{else} \end{array}\right. \end{array}$$ (7)

When the passenger’s arrival time at the platform $\overset{m}{\underset{plat}{t}}$ falls within the scheduled arrival $\overset{arr}{\underset{S_{i}}{t}}$ and departure $\overset{dep}{\underset{S_{i}}{t}}$ time window of train *v*, and the real-time passenger load of train *v* at $\overset{m}{\underset{plat}{t}}$, denoted as $C_{v}(\overset{m}{\underset{plat}{t}})$, is less than 90% of its maximum capacity $\overset{max}{\underset{v}{C}}$, the passenger is allowed to board, in which case $\varphi (\overset{m}{\underset{plat}{t}})$ =1. Otherwise, $\varphi (\overset{m}{\underset{plat}{t}})$ =0, and the passenger remains in the waiting state.

During train operation, the passenger load of the train changes with passenger boarding and alighting, and the variation can be expressed as:


$$\begin{array}{c} C_{v}(t+1)=C_{v}(t)+\overset{up}{\underset{v}{C}}(t)-\overset{down}{\underset{v}{C}}(t) \end{array}$$ (8)

where $C_{v}(t+1)$ and $C_{v}(t)$ are the passenger loads at times t+1 and t, respectively; $\overset{up}{\underset{v}{C}}(t)$ is the number of boarding passengers at time t; and $\overset{down}{\underset{v}{C}}(t)$ is the number of alighting passengers at time t. For transfer scenarios, passengers follow predetermined transfer routes, and the coordination mechanism is reactivated at transfer stations.

### 3.3. Bounded rationality route choice model for passengers under degraded operations

This section develops a bounded rationality route choice model to quantify passengers’ decision-making processes during degraded operations through a multidimensional utility analysis. It further establishes behavioral mechanisms for route reselection based on waiting time thresholds and service degradation levels.

#### 3.3.1. Utility analysis of passenger route choice with bounded rationality under degraded operations.

Under degraded operations in urban rail transit systems, passenger travel decisions exhibit dynamic adjustments. When experiencing prolonged waiting times or emergency-induced service disruptions, passengers demonstrate significantly increased route reselection behavior. Their path choices are not solely determined by objectively optimal routes but are influenced by perceptual biases, cognitive limitations, and subjective preferences [24]. This paper develops a behavioral utility function incorporating four dimensions: time value, transfer burden, degradation impact, and route familiarity.

Research indicates passengers exhibit stronger psychological aversion to “delays” than positive perception of “time savings”, demonstrating asymmetric gain-loss perception [25]. Therefore, we introduce psychological reference time as benchmark and establish the following nonlinear time perception function $U_{t}$:


$$\begin{array}{c} U_{t}=\left\{ \begin{array}{c} (t_{ref}-t_{P_{q}}{)}^{\eta_{t}},t_{P_{q}}\leq t_{ref}\\ -{\mathrm{\backslash Upgamma}}_{t}\cdot (t_{P_{q}}-t_{ref}{)}^{\xi_{t}},t_{P_{q}}>t_{ref} \end{array}\right. \end{array}$$ (9)

Where $t_{P_{q}}$ denotes the estimated travel time of route $P_{q}$, $t_{ref}$ represents the passenger’s psychological reference time, $\eta_{t}$ and $\xi_{t}$ being the sensitivity coefficients for time savings and delays respectively, and ${\mathrm{\backslash Upgamma}}_{t}$ standing for the delay aversion coefficient. When $t_{P_{q}}\leq t_{ref}$, passengers experience positive utility due to perceived time savings, whereas when $t_{P_{q}}>t_{ref}$, the utility becomes negative. A higher $U_{t}$ value reflects better temporal acceptance of route $P_{q}$, while a lower value indicates poorer acceptance.

Furthermore, transfer behavior typically involves spatial navigation, station wayfinding, stairway movements, and waiting uncertainty [26], which can significantly exacerbate travel stress particularly during peak hours or under station congestion conditions. A transfer burden term is formulated to characterize passenger utility:


$$\begin{array}{c} U_{H}=-\lambda_{H}\cdot H_{P_{q}}\cdot (1+\zeta_{H}\cdot \rho_{S_{i}}(t)) \end{array}$$ (10)

Where $H_{P_{q}}$ denotes the number of transfers along the route, $\rho_{S_{i}}(t)$ represents the passenger flow density at transfer station $S_{i}$, $\lambda_{H}$ indicates the base transfer penalty factor, and $\zeta_{H}$ stands for the crowding sensitivity parameter, the absolute value of $U_{H}$ quantitatively reflects passengers’ aversion degree to transfer pressure, with larger values corresponding to stronger aversion.

The impact of operational degradation on passenger route choice is predicated on a fundamental premise: passengers must first acquire degradation information before it can influence their route utility [27,28]. To characterize this “perception-before-influence” decision mechanism, the following degradation utility function is formulated:


$$\begin{array}{c} U_{L}=-\lambda_{L}\cdot L_{\omega } \end{array}$$ (11)

Where $L_{\omega }$ represents the objective level of operational degradation with higher values indicating greater severity, and $\lambda_{L}$ denotes passenger *m*’s degradation perception coefficient within the range [0,+∞) quantifying heterogeneity in information asymmetry environments, the absolute value of $U_{L}$ directly characterizes degradation’s inhibitory effect on route selection. When $\lambda_{L}$ =0, passengers remain uninfluenced by degradation data; when 0<$\lambda_{L}$ ≤1, partial information acquisition occurs; when $\lambda_{L}$ >1, psychological factors like anxiety may cause overestimation of degradation impacts, with larger absolute $U_{L}$ values indicating stronger route selection inhibition.

To characterize passengers’ preference for familiar routes in path selection, route familiarity is incorporated as a component of the utility function [29]. Considering its diminishing marginal utility characteristic, a logarithmic function is adopted for modeling as follows:


$$\begin{array}{c} U_{F}=\lambda_{F}\cdot \ln(F_{P_{q}}+1) \end{array}$$ (12)

Where $F_{P_{q}}$ denotes the passenger’s familiarity level with route $P_{q}$, $\lambda_{F}$ represents the familiarity utility coefficient modulating its contribution to the composite utility function, and $U_{F}$ quantifies the utility increment attributable to route familiarity. The logarithmic specification captures the fundamental property of diminishing marginal returns, manifesting as a progressive deceleration in utility enhancement with increasing familiarity levels. This formulation embodies the cognitive stability phenomenon wherein elevated familiarity can override temporal or transfer-related disadvantages in route selection. The utility progression is principally governed by the accumulated frequency of route utilization, with the logarithmic transformation ensuring mathematical rigor in representing the characteristic monotonic yet concave relationship.

Based on the aforementioned analysis, the comprehensive perceived utility of route $P_{q}$ for passenger m can be expressed as:


$$\begin{array}{c} U_{m}(P_{q})=U_{t}-U_{H}-U_{L_{\omega }}+U_{F} \end{array}$$ (13)

Where $U_{m}(P_{q})$ denotes the comprehensive perceived utility of route $P_{q}$ for passenger *m*, higher utility values indicate stronger route preference, while lower values reflect diminished selection propensity due to more pronounced adverse impacts.

#### 3.3.2. Bounded rationality route choice model for passengers under degraded operations.

Under degraded operations, the primary driver for passengers’ route reselection behavior is the significant decline in their subjectively perceived route utility. The route reselection behavior of passenger m is mainly triggered by the following two conditions: First, when the cumulative waiting time $\overset{m}{\underset{\text{wait}}{t}}$ of the passenger on the platform exceeds their individually acceptable maximum waiting time threshold $\overset{m}{\underset{\text{delay}}{t}}$, the condition is satisfied:


$$\begin{array}{c} \overset{m}{\underset{\text{wait}}{t}}\geq \overset{m}{\underset{1}{\gamma }}\cdot \overset{m}{\underset{\text{delay}}{t}} \end{array}$$ (14)

Where $\overset{m}{\underset{1}{\gamma }}$ denotes passenger m’s waiting time tolerance coefficient reflecting individual sensitivity to delays, and $\overset{m}{\underset{\text{delay}}{t}}$ represents the maximum acceptable waiting time threshold obtained through travel surveys using questionnaire and observational methods to determine context-dependent tolerance levels across passenger types.

Secondly, the trigger condition based on perceived degradation impact. When the subjectively perceived negative impact intensity of degradation (i.e., the absolute value of degradation utility $\left|U_{L}\right|$) exceeds an individual tolerance threshold $\overset{m}{\underset{L,tol}{U}}$, it prompts the passenger to reevaluate their route choice. The specific trigger condition is:


$$\begin{array}{c} |U_{L}|\geq \overset{m}{\underset{2}{\gamma }}\cdot \overset{m}{\underset{L,tol}{U}} \end{array}$$ (15)

Where $\overset{m}{\underset{2}{\gamma }}$ denotes the tolerance coefficient for degraded service levels, representing passenger m’s acceptance threshold for degraded operations encountered on their current route.

When route reselection conditions are satisfied, passengers proceed to reevaluate their route choices based on real-time operational conditions. In addition to selecting alternative physical routes, passengers may also opt to abandon their journey or switch to other transportation modes. Such behavioral adaptations can be formally modeled as a “virtual path” selection $P_{0}$, with its corresponding utility function formulated as:


$$\begin{array}{c} \overset{m}{\underset{P_{0}}{U}}=-(\gamma_{1}\cdot max(0,\overset{m}{\underset{wait}{t}}-\overset{m}{\underset{delay}{t}})+\gamma_{2}\cdot max(0,|U_{L}|-\overset{m}{\underset{L,tol}{U}})) \end{array}$$ (16)

When any triggering condition is met, passengers will reassess candidate route sets and select alternative routes based on bounded rationality principles [30]. The route selection process is described using a Logit model, where the probability of passenger m choosing route $\overset{\prime }{\underset{q}{P}}$ is given by the following formula:


$$\begin{array}{c} X_{m}(\overset{\prime }{\underset{q}{P}})=\frac{e^{\mu_{m}\cdot U_{m}(\overset{\prime }{\underset{q}{P}})}}{\sum_{q=0}^{Q}e^{\mu_{m}\cdot U_{m}(\overset{\prime }{\underset{q}{P}})}} \end{array}$$ (17)

Where P′ denotes the set of available routes after reselection, with $\overset{\prime }{\underset{q}{P}}$ representing the q-th route in this set, the numerator $e^{\mu_{m}\cdot U_{m}(\overset{\prime }{\underset{q}{P}})}$ quantifies the exponentially scaled perceived utility of route $\overset{\prime }{\underset{q}{P}}$ for passenger m, reflecting route preference intensity through the rationality coefficient $\mu_{m}$. The denominator $\sum_{q=0}^{Q}e^{\mu_{m}\cdot U_{m}(\overset{\prime }{\underset{q}{P}})}$ normalizes the probabilistic choice structure by aggregating weighted utilities across all candidate routes. Given cognitive instability under service disruptions, the rationality coefficient is formulated as a dynamic parameter influenced by disturbance factors, with its variation expressed through:


$$\begin{array}{c} \mu_{m}=\mu_{0}+\mu_{1}\cdot \overset{m}{\underset{\text{wait}}{t}}+\mu_{2}\cdot |U_{L}| \end{array}$$ (18)

Where $\mu_{0}$ denotes the baseline rationality level of passenger m, with $\mu_{1}$ and $\mu_{2}$ representing the influence coefficients of waiting time and operational mode degradation on rationality respectively, the model ultimately generates a choice probability vector $X_{m}$ quantifying passenger m’s selection preferences across all available routes in the remaining set P′:


$$\begin{array}{c} X_{m}=[X_{m}(\overset{\prime }{\underset{0}{P}}),X_{m}(\overset{\prime }{\underset{1}{P}}),X_{m}(\overset{\prime }{\underset{2}{P}})\ldots ,X_{m}(\overset{\prime }{\underset{Q}{P}})] \end{array}$$ (19)

## 4. Passenger-train coordinated passenger flow propagation model with dynamic route choice under degraded operations

Under degraded operations in urban rail transit systems, localized passenger accumulation tends to occur at specific stations due to service capacity reduction, operational constraints, or passenger behavioral adaptation. Such localized congestion progressively propagates pressure to other network nodes through passenger flow movement and route selection behaviors. To quantitatively characterize the formation, evolution, and propagation mechanisms of passenger flow aggregation in station and network environments, we develop a propagation model [31].

At any time t during degraded operations, the total passenger count $N_{S_{i}}(t)$ at station $S_{i}$ comprises stranded passengers $\overset{stay}{\underset{S_{i}}{N}}(t)$, newly entering passengers $\overset{entry}{\underset{S_{i}}{N}}(t)$, alighting passengers $\overset{alight}{\underset{S_{i}}{N}}(t)$, boarding passengers $\overset{board}{\underset{S_{i}}{N}}(t)$, and diverted passengers $\overset{loss}{\underset{S_{i}}{N}}(t)$, formulated as:


$$\begin{array}{c} N_{S_{i}}(t)=\overset{stay}{\underset{S_{i}}{N}}(t)+\overset{entry}{\underset{S_{i}}{N}}(t)+\overset{alight}{\underset{S_{i}}{N}}(t)-\overset{board}{\underset{S_{i}}{N}}(t)-\overset{loss}{\underset{S_{i}}{N}}(t) \end{array}$$ (20)

The diverted passenger count $\overset{loss}{\underset{S_{i}}{N}}(t)$ can be calculated by integrating the output of the route reselection model [32]. This value quantifies the passenger flow loss resulting from passengers altering their routes and no longer passing through the station. The calculation formula is expressed as:


$$\begin{array}{c} \overset{\text{loss}}{\underset{S_{i}}{N}}(t)=\sum_{m\in M_{S_{i}}}\sum_{\begin{array}{c} \overset{\prime }{\underset{q}{P}}\in P\\ S_{i}\notin \overset{\prime }{\underset{q}{P}} \end{array}}X_{m}\left(\overset{\prime }{\underset{q}{P}}\right) \end{array}$$ (21)

Where $M_{s_{i}}$ denotes the set of passengers whose original routes contain station $S_{i}$ at time t, $X_{m}(\overset{\prime }{\underset{q}{P}})$ indicates passenger m’s selection probability for new route $\overset{\prime }{\underset{q}{P}}$, the inner summation calculates diversion probability by accumulating selection probabilities for all routes excluding $S_{i}$, while the outer summation aggregates expected diversion quantities across all affected passengers to determine the total diverted passenger count at station $S_{i}$.

Building upon the established rail network structure, to further characterize passenger flow aggregation and propagation mechanisms under degraded operations, the passenger flow density at station $S_{i}$ during any degraded operations time t is defined as:


$$\begin{array}{c} \rho_{S_{i}}(t)=\frac{N_{S_{i}}(t)}{C_{S_{i}}} \end{array}$$ (22)

Where $N_{S_{i}}(t)$ denotes the in-station passenger count and $C_{S_{i}}$ represents the station’s design capacity, the passenger flow density is mathematically defined.

Under scenarios without dispatch intervention or evacuation mechanisms, stations experiencing passenger accumulation will propagate passenger flow pressure to adjacent stations through topological connections and route networks [33]. The passenger density increment imposed by station $S_{i}$ on neighboring station $S_{j}$ ∈ S is defined as:


$$\begin{array}{c} \mathrm{\backslash Updelta}\rho_{S_{j}}(t+1)=\tau_{e_{ij}}\cdot \rho_{S_{i}}(t) \end{array}$$ (23)

Where $\tau_{e_{ij}}$ represents the propagation coefficient quantifying inter-station transmission intensity, this parameter is formulated through route overlap analysis incorporating both fundamental edge weights and route selection probability adjustments, defined as:


$$\begin{array}{c} \tau_{e_{ij}}=\sigma \cdot g_{e_{ij}}\cdot \left(1+\frac{1}{\left|M_{e_{ij}}\right|}\sum_{m\in M_{e_{ij}}}X_{m}\left(\overset{\prime }{\underset{q}{P}}\right)\right) \end{array}$$ (24)

Where σ denotes the global propagation adjustment factor regulating system-wide transmission intensity, $g_{e_{ij}}$ represents the fundamental propagation weight of edge $e_{ij}$ determined by inter-station distance and connectivity strength, and $M_{e_{ij}}$ indicates the passenger set traversing from $S_{i}$ to $S_{j}$ through route $\overset{\prime }{\underset{q}{P}}$, the net passenger flow $\eta_{S_{j}}(t)$ is introduced to characterize station density dynamics, defined as the net number of passengers entering station $S_{j}$ per unit time:


$$\begin{array}{c} \eta_{S_{j}}(t)=\sum_{m\in M_{S_{j}}}X_{m}(\overset{\prime }{\underset{q}{P}})\cdot \left[\overset{\text{enter}}{\underset{S_{j}}{N}}(t)-\overset{\text{exit}}{\underset{S_{j}}{N}}(t)\right] \end{array}$$ (25)

Where $\overset{\text{enter}}{\underset{S_{j}}{N}}(t)$ denotes the total passenger inflow at station $S_{j}$ during time *t*, $\overset{\text{exit}}{\underset{S_{j}}{N}}(t)$ represents the corresponding passenger outflow, and $M_{S_{j}}$ constitutes the complete set of passengers associated with station $S_{j}$, the passenger flow density at station $S_{j}$ at time t + 1 is formulated by integrating propagation effects from adjacent nodes with the station’s net passenger flow input:


$$\begin{array}{c} \rho_{S_{j}}(t+1)=\rho_{S_{j}}(t)+\sum_{i,j\in S}\tau_{e_{ij}}\cdot \rho_{S_{i}}(t)+\frac{\eta_{S_{j}}(t)}{C_{S_{j}}} \end{array}$$ (26)

Where $\rho_{S_{j}}(t)$ denotes the passenger flow density at station $S_{j}$ at time t, and $\sum_{i,j\in S}\tau_{e_{ij}}\cdot \rho_{S_{i}}(t)$ represents the aggregate passenger flow density transmitted from adjacent stations through topological connections.

To characterize the passenger flow propagation effect between stations, the propagation intensity from station $S_{i}$ to adjacent station $S_{j}$ at time t is defined as:


$$\begin{array}{c} Z_{e_{ij}}(t)=\tau_{e_{ij}}\cdot \rho_{S_{i}}(t) \end{array}$$ (27)

Propagation intensity is used to characterize the degree to which passenger flow pressure is transmitted between stations, reflecting the diffusion capability of congestion within the network structure. When the propagation intensity approaches 0, congestion is largely confined to a single station, with limited influence on adjacent stations. When it falls within the range of 0.3–0.5, passenger flow pressure begins to spread to neighboring stations. When the value exceeds 0.7, congestion exhibits significant cross-station propagation characteristics, indicating strengthened inter-station coupling and an increased risk of regional congestion diffusion. When propagation intensity approaches 0.8 or higher, the system is typically considered to be in a high propagation risk stage, where congestion pressure at core stations is clearly transmitting to surrounding stations, potentially leading to broader operational instability.

## 5. Results

This chapter designs multiple sets of controlled experiments based on actual data from the Hangzhou Metro, focusing on empirical analysis across three dimensions: path recognition accuracy, reselection behavior response patterns, and dynamic characteristics of passenger flow propagation.

### 5.1. Research areas and data

**Fig 4:**
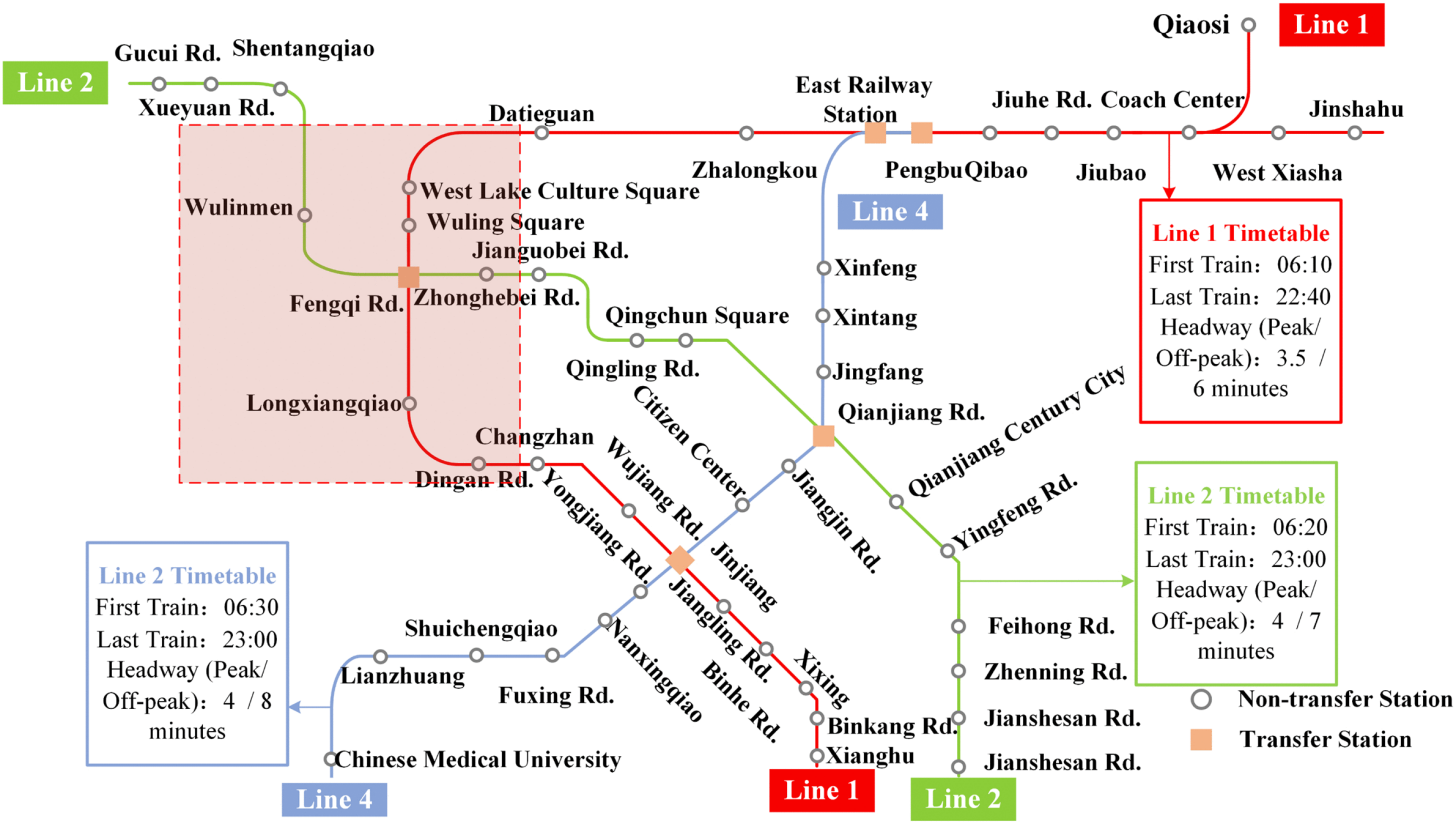
Network topology and operating timetables of Hangzhou Metro Lines 1, 2, and 4.

This study focuses on Hangzhou Metro Lines 1, 2, and 4, with the network consisting of 80 stations, 5 transfer stations, and 27 interlocking areas, showcasing the typical features of an urban rail transit network. The network topology and the operating timetables of each line are shown in Fig 4.

The dataset employed includes AFC data collected over a 15-day period in January 2019, encompassing 19,898,175 valid travel records with a daily average of 1,326,545 records. Each AFC record contains crucial information, such as anonymized user ID, entry and exit stations, and timestamps. Table 1 presents the basic structure of the AFC data.

**Table 1:** Sample AFC Data of Hangzhou Metro.

userID	start_line	start_station	start_ time	end_line	end_station	end_ time
Baecfa51276fa6f1e432963c4cb3f6a0f	C	52	2019/1/106:00	C	36	2019/1/106:43
Da2260acda93095e55bf466b8993d58b5	B	13	2019/1/109:33	B	5	2019/1/109:55
Bb8e687d5aedc9040879b67952787c35d	B	15	2019/1/112:38	C	66	2019/1/113:41
C03b90c5dfab9ac10a00e2fca9ac7aa11	B	15	2019/1/117:58	B	24	2019/1/118:28

Table 2 provides the arrival and departure times of trains at each station. When integrated with the AFC dataset, these records serve as a temporal reference for identifying individual passenger travel paths.

**Table 2:** Example of Train Timetable on Hangzhou Metro Line 1.

Train Number	Station Name	Arrival Time	Departure Time	Dwell Time (s)
DT1001	West Lake Culture Square	07:00:15	07:00:45	30
DT1001	Wulin Square	07:03:20	07:03:50	30
DT1001	Fengqi Rd.	07:06:10	07:06:40	45

### 5.2 Questionnaire survey for behavioral parameters

A questionnaire survey was conducted to obtain and determine the value range of the waiting time tolerance threshold in the bounded rationality model.

The eligible participants for the survey were urban rail transit users aged 18 and above who had prior experience using metro systems. Participants were required to have taken metro trips at least twice in the past month to ensure sufficient familiarity with waiting time perception and route choice behavior.

Participants were selected using a convenience sampling strategy combined with voluntary participation. The survey was conducted entirely offline and administered face-to-face in urban rail transit stations. Respondents completed the questionnaire on-site during their trips, ensuring that they had direct and recent travel experience relevant to the study context.

The survey was conducted in Lanzhou, Gansu Province, China. The questionnaire survey was conducted on April 15 and April 17, 2025, during typical weekday conditions to reflect regular passenger travel behavior.

The waiting time tolerance threshold was determined based on the questionnaire responses regarding acceptable waiting time under typical travel conditions. Specifically, the distribution of acceptable waiting time reported by respondents was statistically analyzed, and the corresponding tolerance coefficient range was calibrated accordingly. The final parameter range was set within [0.6, 0.8], ensuring consistency with both empirical survey results and established findings in the literature. This calibrated range was subsequently used in the model parameter settings.

### 5.3 Experimental scenarios and parameter settings

The path generation and identification experiment relies on properly defined parameters, which are first calibrated from data. By statistically analyzing the difference between the actual travel times of transfer passengers in the AFC dataset and train operation times, the walking time coefficient is determined to be 0.8. After calibrating the passenger route choice behavior model using the maximum likelihood estimation method, the transfer penalty coefficient is set to 1.5.

An experimental scenario is then constructed for the morning peak period (07:00–09:00), focusing on the interlocking area between West Lake Cultural Square and Fengqi Road. To reflect operating conditions under degraded operations, different degraded operations and corresponding control strategies and headways are considered, as shown in Table 3.

**Table 3:** Control modes and headways under degraded operations.

system degradation scenarios	Control mode	Headway (min)
L_1_	Automatic driving under ATP protection (no ATO)	8
L_2_	Manual driving with fixed block (ITC mode)	12
L_3_	Full manual driving (interlocking control)	16

It should be emphasized that the proposed simulation framework is established on the complete network topology of Hangzhou Metro Lines 1, 2, and 4. Path generation, trajectory identification, and route reselection are all conducted over the full candidate path set across the entire network. Consequently, under degraded operation scenarios, passengers are allowed to select alternative routes involving cross-line transfers and network-wide detours. The present study concentrates on the interlocking section with the most pronounced degradation impact for result presentation, as this area exhibits the most significant passenger accumulation and behavioral adaptation. Such a localized analytical focus is adopted to clearly demonstrate the core propagation mechanisms triggered by degraded operations, while the underlying computation and route choice processes remain network-wide.

During the parameterization of the bounded rational route reselection model, this study adopts a systematic calibration framework that integrates data-driven analysis with evidence from the existing literature. This approach ensures that the model is both empirically grounded and theoretically consistent with established behavioral theories. The parameters are categorized into three groups: operational time-related parameters, behavioral perception parameters, and key decision-making parameters. Each group is estimated using a targeted calibration strategy.

First, parameters related to path identification and operational travel time are jointly estimated using AFC data and train timetables. By matching observed travel times from AFC records with theoretically calculated travel times derived from timetables, the distribution of time deviations is obtained. An optimization model is then constructed with the objective of minimizing the Mean Squared Error (MSE) between observed and estimated travel times, and the walking time coefficient is calibrated accordingly. Through statistical optimization, the walking time coefficient is determined such that the model-estimated travel times are statistically consistent with observed values, thereby improving the accuracy of path identification and the reliability of travel time representation.

Second, behavioral perception parameters—including the time sensitivity coefficient, delay aversion coefficient, and rationality coefficient—are primarily determined based on empirically validated parameter ranges reported in prior studies on rail transit route choice behavior and prospect theory-related research [27–33]. These parameter ranges are further constrained to ensure compatibility with the bounded rational decision-making framework developed in this study. Such parameters characterize passengers’ psychological perception intensity under delay conditions, risk-averse tendencies, and bounded rationality attributes. The adopted intervals ensure consistency with established empirical findings while preventing parameter values that would deviate from recognized behavioral regularities.

Finally, key behavioral parameters that directly influence the probability distribution of route choice—such as the transfer penalty coefficient and the degradation sensitivity coefficient—are calibrated using a likelihood-based estimation approach grounded in observed data. Specifically, the actual route choice distribution is constructed based on AFC-based path identification results. The route choice probabilities generated by the model are then compared with the observed distribution, and the log-likelihood function is employed as the goodness-of-fit metric. A discrete parameter search is conducted within predefined feasible intervals, and the parameter combination that maximizes the log-likelihood value is selected as the final estimate, thereby completing the calibration of the key behavioral parameters.

Based on the above statistical analysis and optimization procedures, the calibrated parameter ranges of the model are summarized in Table 4.

**Table 4:** Parameter settings of the bounded rational route re-selection model.

Parameter category	Symbol	Range	Source
Passenger psychological reference time	*t*_*ref*_	[1.0*t*_*0*_,1.5 *t*_*0*_]	Literature [27,30]
Cost sensitivity coefficient	*η*_*t*_	[1.2,1.8]	Literature [28,33]
Delay sensitivity coefficient	*ξ*_*t*_	[0.3,1]	Literature [27,29]
Delay aversion coefficient	Γ_*t*_	[1.5,2.5]	Literature [30,33]
Basic transfer penalty factor	*λ*_*H*_	[0,2]	Model calibration
Congestion sensitivity parameter	*ζ*_*H*_	[0.1,0.8]	AFC data analysis
Sensitivity to degraded operation	*λ*_*L*_	[0,1]	Model calibration
Degradation impact parameters	*L*_*ω*_	[1,2,3]	Operational rules and specifications [26,31]
Familiarity utility gain factor	*λ*_*F*_	[0,1]	AFC statistical calculation
Route familiarity adjustment factor	*ξ*_*L*_	[0.5,0.7]	Operational delay statistics
Passenger waiting time tolerance	* $\overset{m}{\underset{1}{\gamma }}$ *	[0.6,0.8]	Questionnaire survey and literature [27,32]
Tolerance for degraded operation	$\overset{m}{\underset{2}{\gamma }}$	[0.4,0.6]	Literature [31,32]
Rationality coefficient	*μ*_*m*_	[14,16] min	Literature [23,28,32]

Finally, a parameter system is established in the passenger flow propagation model to characterize the dynamic evolution of passenger flows under degraded operations. Based on Eqs. (22)–(27), the key parameter settings are as follows: station capacity is set to 2,500 passengers for transfer stations and 1,800 passengers for ordinary stations; the global adjustment factor of the propagation coefficient is within [0.5, 0.8], the basic edge weight within [0.1, 0.5]; and in computing net passenger flow, the path choice probability is dynamically generated by the bounded rational model.

### 5.4. Experimental results

Based on the parameter settings in the Experimental Scenarios and Parameter Settings sections, the path generation algorithm ultimately generates 2,876 valid paths. The path identification simulation is then conducted using the generated paths, and the results are shown in Table 5.

**Table 5:** Statistics of Path Identification Results Based on AFC Data.

Data Type	Record Count	Recognition Success Count	Recognition Rate
Workday Data	13,265,450	12,759,400	96.2%
Weekend Data	6,632,725	6,263,103	94.3%
Direct Travel	14,228,722	13,958,371	98.1%
Transfer Travel	5,669,453	5,064,132	91.3%
Peak Hours	9,949,088	9,423,101	94.7%
Off-Peak Hours	9,949,087	9,599,402	96.5%

**Fig 5:**
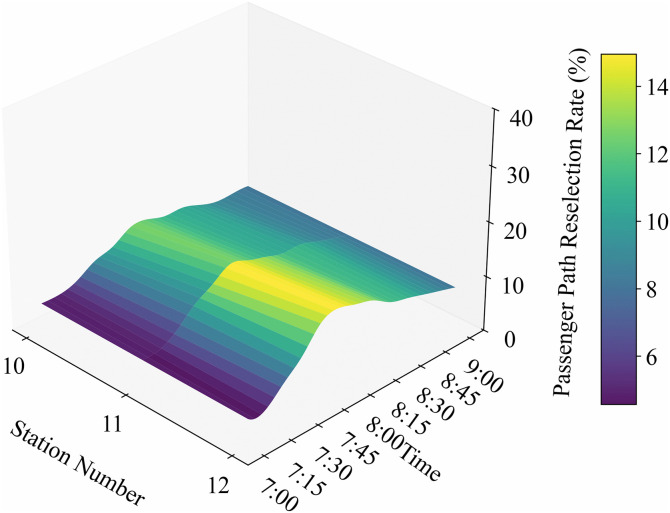
Passenger Path Re-Selection Rates at Stations in the Interlocking Area Under L1 Degraded Operations.

**Fig 6:**
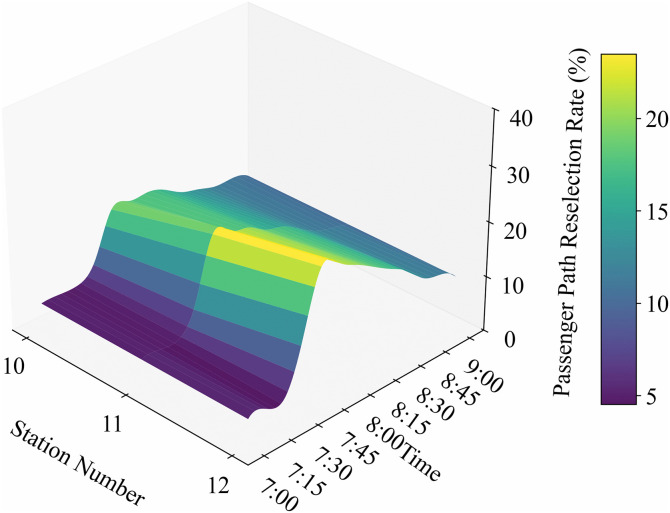
Passenger Path Re-Selection Rates at Stations in the Interlocking Area Under L2 Degraded Operations.

**Fig 7:**
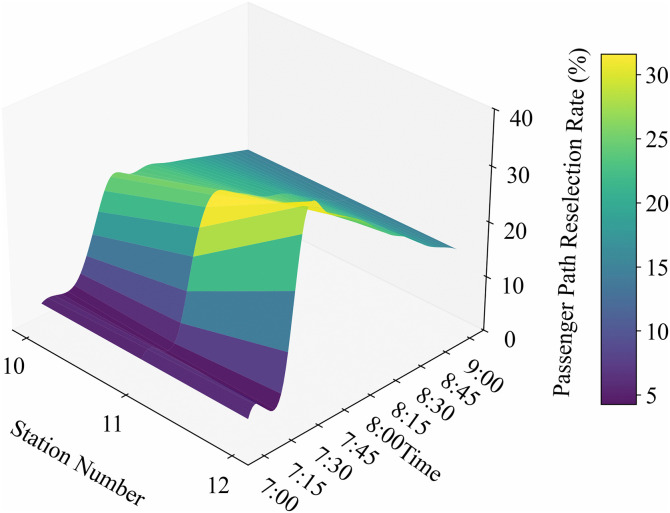
Passenger Path Re-Selection Rates at Stations in the Interlocking Area Under L3 Degraded Operations.

In the context of the experimental scenarios and parameter settings mentioned above, this study systematically simulates passenger path re-selection behavior under degraded operations, with the simulation results presented as follows:

As shown in Figs 5–7, the path re-selection rate at West Lake Cultural Square Station, Wulin Rd. Station, and Fengqi Rd. Station increases with the severity of degraded operations (L1, L2, L3), with Fengqi Rd. Station exhibiting the highest rate, followed by Wulin Rd. Station, and West Lake Cultural Square Station showing the lowest. To further illustrate this trend, Table 6 compares the peak path re-selection rates of passengers at stations within the degraded operations interlocking area under different degraded operations.

**Table 6:** Peak Proportion of Passenger Path Re-selection in the Interlocking Area under Different Degraded operations.

Station	L1 (Bounded Rationality)	L1 (Fully Rational)	L2 (Actual Value)	L2 (Bounded Rationality)	L2 (Fully Rational)	L3 (Bounded Rationality)	L3 (Fully Rational)
West Lake Cultural Square	12.5%	21.4%	19.8%	18.9%	32.7%	24.3%	45.8%
Wulin Rd.	15.8%	24.6%	24.2%	23.6%	36.9%	31.6%	50.2%
Fengqi Rd.	18.3%	27.3%	29.4%	28.1%	41.5%	38.2%	56.4%

Table 6 shows that as the severity of degraded operations increases, the peak path re-selection rate at stations within the interlocking area progressively rises. Specifically, the peak re-selection rate at Fengqi Rd. Station increases from 18.3% under L1 to 28.1% under L2, and further to 38.2% under L3, while Wulin Rd. Station and West Lake Cultural Square Station exhibit similar trends. This highlights the significant impact of degraded operations changes on passenger path choices, with passengers more likely to switch routes as degraded operations intensify.

To compare the consistency between model results under different rationality assumptions and actual observations, passenger route reselection rates under the fully rational and bounded rationality settings were first compared. On this basis, the observed values under the L2 degraded operation scenario were selected as the reference benchmark for further analysis. The results indicate that under all degraded operation levels, the fully rational model yields higher route reselection proportions than the bounded rationality model, and the gap between the two models widens as the degradation level increases. The difference intervals between the two model results under L1, L2, and L3 are [8.8%, 9.0%], [13.3%, 13.8%], and [18.2%, 21.5%], respectively.

Under the L2 degraded scenario, the results of both rationality assumptions were further compared with the actual observed values. At West Lake Cultural Square Station, the deviations of the bounded rationality and fully rational models are 0.9% and 12.9%, respectively; at Wulin Rd. Station, they are 0.6% and 12.7%; and at Fengqi Rd. Station, they are 1.3% and 12.1%. Correspondingly, the deviation range of the bounded rationality model is 0.6%–1.3%, whereas that of the fully rational model is 12.1%–12.9%. A clear disparity in deviation magnitude is observed between the two models, with the bounded rationality model exhibiting substantially smaller numerical deviations from the actual observations overall.

To further examine the impact of path re-selection on passenger flow distribution, a typical scenario is analyzed where the degraded operations shifts from normal to L2. The focus is on passenger flow changes at stations within the interlocking area and their neighbors during the morning peak, with results shown in Table 7.

**Table 7:** Passenger Flow Comparison between Interlocking Area Stations and Adjacent Stations under L2 degraded operations (7:00–9:00).

Station	Normal Flow	Degraded Flow	Route Re-selection (%)	Count	Abandonment (%)
Datieguan Station	2236	2320	−3.76%	116	1.20%
Zhanongkou Station	2704	2816	−4.14%	76	1.30%
West Lake Cultural Square Station	3185	2582	18.93%	−507	3.00%
Wulin Rd. Station	2763	2110	23.63%	−524	4.60%
Fengqi Rd. Station	2472	1778	28.07%	−536	6.40%
Longxiang Qiao Station	3845	4172	−8.50%	269	1.50%
Dingan Rd. Station	3028	3295	−8.82%	233	1.10%

As shown in Table 7, under the L2 degraded operations, passenger flow within the interlocking area decreases significantly, while flow at forward adjacent stations rises notably. For example, Datieguan Station sees a 3.76% increase, whereas West Lake Cultural Square Station experiences an 18.93% decrease. This suggests that most passengers re-select alternative paths to bypass the affected area, while a smaller proportion abandon their trips. Consequently, passenger flow shifts from the interlocking area to the forward adjacent stations, highlighting the significant influence of passenger behavior on regional flow distribution.

**Fig 8:**
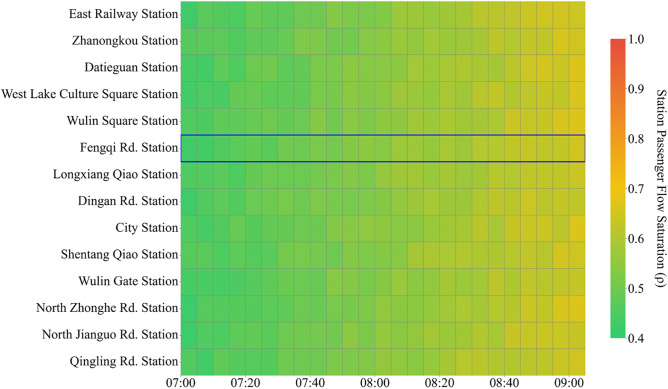
Passenger Flow Evolution Heatmap of Core Stations and Their Adjacent Stations Under Normal Operating Conditions.

**Fig 9:**
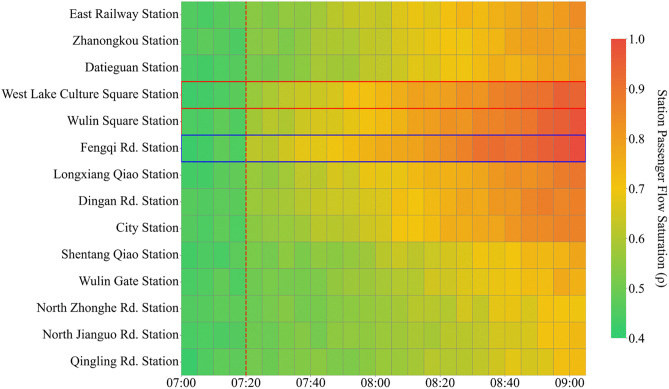
Passenger Flow Evolution Heatmap of Core Stations and Their Adjacent Stations Under L1 Degraded Operations.

**Fig 10:**
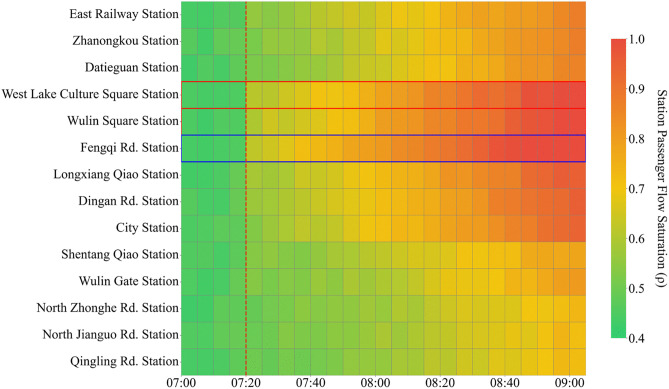
Passenger Flow Evolution Heatmap of Core Stations and Their Adjacent Stations Under L2 Degraded Operations.

**Fig 11:**
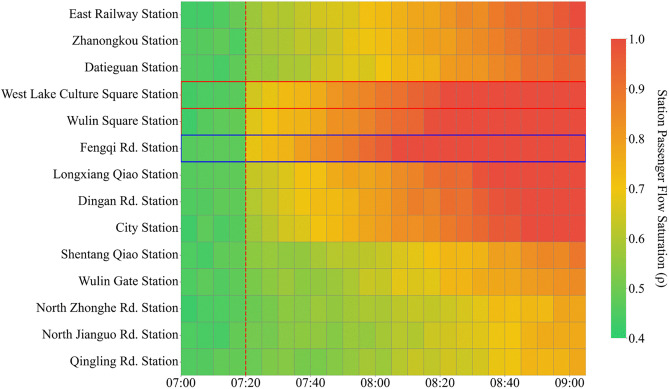
Passenger Flow Evolution Heatmap of Core Stations and Their Adjacent Stations Under L3 Degraded Operations.

To intuitively demonstrate the impact of converting an interlocking area station to an degraded operations on passenger flow propagation, this study conducted simulation experiments using the integrated passenger-train flow propagation model with dynamic path choice. Station saturation heatmaps under L1 to L3 degraded operations were generated and compared with the flow evolution heatmap under normal conditions, as shown in Figs 8–11.

As shown in Figs 8–11, under normal operating conditions, the overall passenger flow saturation at stations remains below 0.7 and is evenly distributed. Once the system enters degraded operations, saturation at core stations increases significantly, particularly at Fengqi Rd. Station and West Lake Cultural Square Station. In the L1 mode, passenger flow concentration is confined to core stations; under L2, the concentration intensifies and gradually spreads to adjacent stations; and under L3, high-saturation areas emerge at both core and adjacent stations, leading to extended congestion. Overall, the pattern evolves from balanced distribution to core concentration, adjacent diffusion, and regional congestion, with higher modes expanding the scope and duration of congestion.

**Fig 12:**
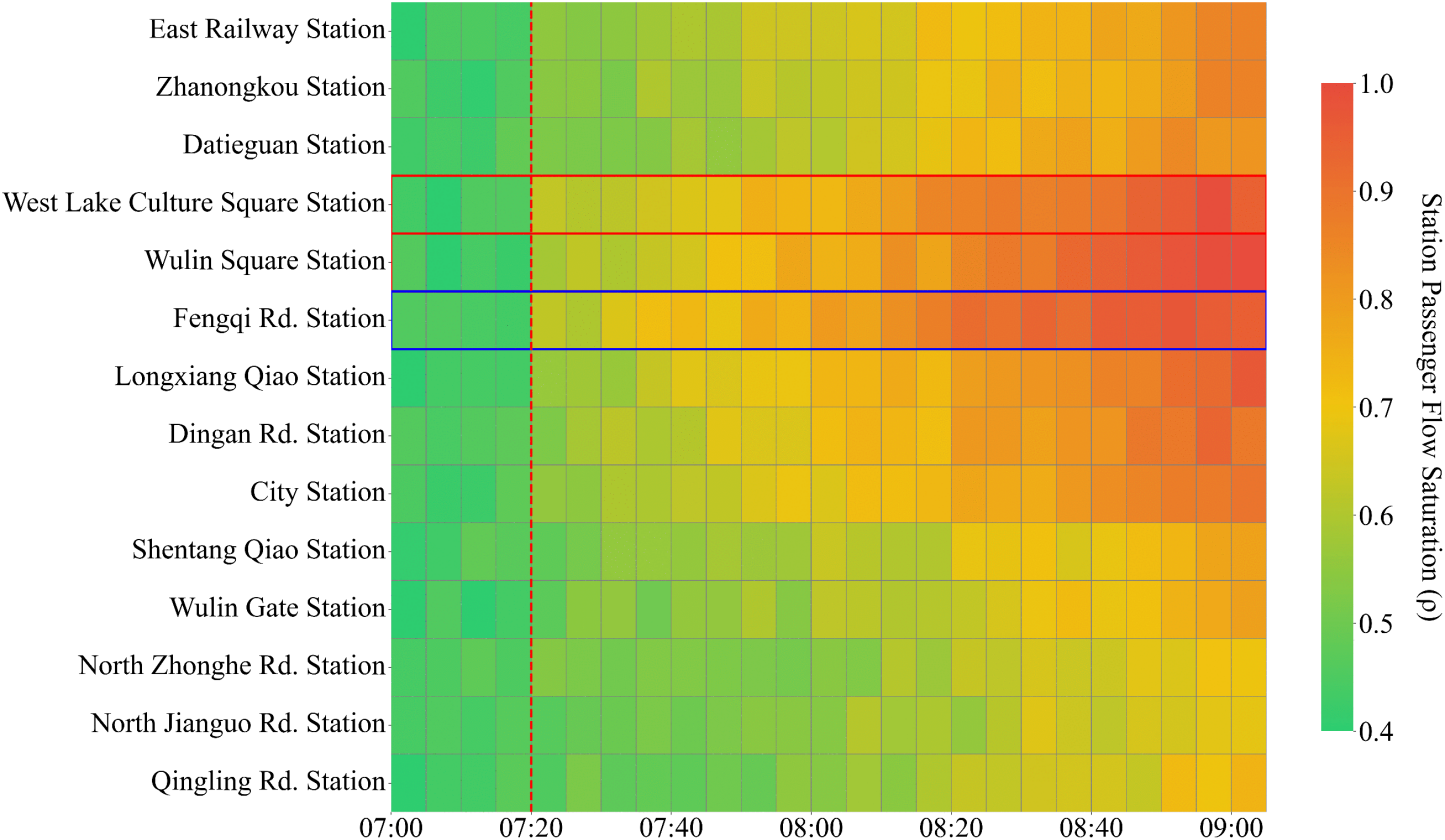
Observed Passenger Flow at Core Stations Under L2 Degraded Operations.

To evaluate the discrepancy between the simulation results and actual operational data under the L2 degraded operation scenario, a point-by-point comparison was conducted using 350 “station–time” sample points derived from 14 stations and 25 time intervals within the core section affected by the degradation. The calculated performance metrics are as follows: RMSE = 0.021, MAE = 0.016, MRE = 3.8%, and coefficient of determination R^2^ = 0.93.Fig 12 presents the spatiotemporal evolution of observed station passenger flow density under the L2 degraded operation scenario. In terms of spatiotemporal distribution characteristics, the simulation results exhibit a high degree of consistency with the observed data with respect to the upward density trend at core stations, the direction of diffusion, and the timing of growth inflection points. Both display an evolutionary pattern characterized by gradual diffusion from the core area to adjacent sections, with closely aligned overall trends.

**Fig 13:**
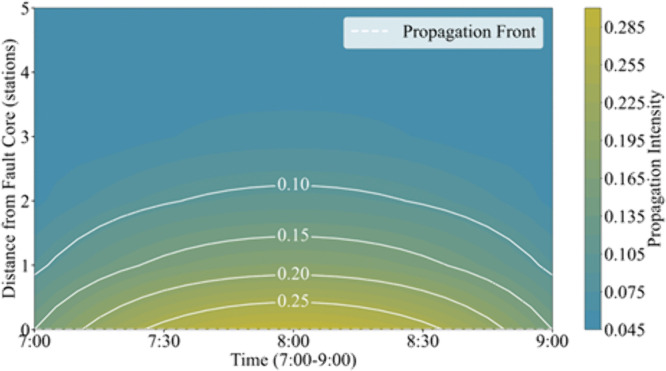
Spatiotemporal Distribution of Passenger Flow Propagation Intensity Under L1 Degraded Operations.

**Fig 14:**
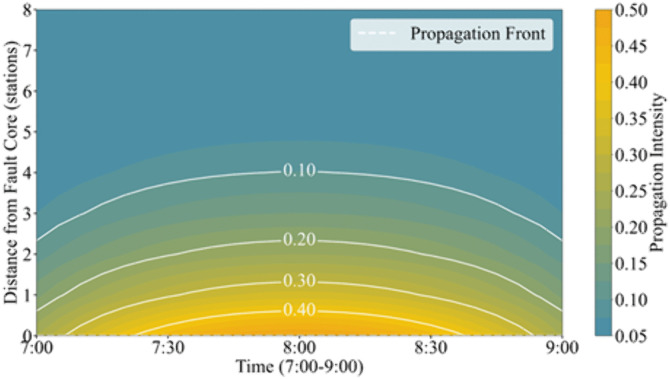
Spatiotemporal Distribution of Passenger Flow Propagation Intensity Under L2 Degraded Operations.

**Fig 15:**
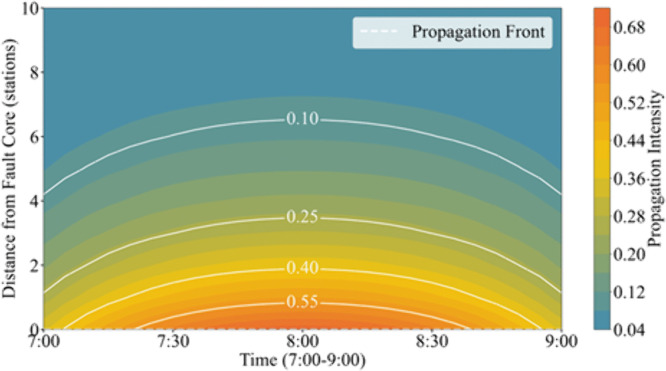
Spatiotemporal Distribution of Passenger Flow Propagation Intensity Under L3 Degraded Operations.

The analysis above shows that as the severity of degraded operations increases, the concentration of passenger flow at stations intensifies, expands, and persists for longer durations. To further examine the spatiotemporal propagation characteristics, Figs 13–15 illustrates the intensity of passenger flow propagation under L1 to L3 degraded operations, based on the simulation results of the passenger flow propagation model.

As shown in Figs 13–15, passenger flow propagation intensifies as the degraded operations de-escalates from L1 to L3. The intensity evolves from localized concentration under L1 to widespread diffusion under L3, with the affected area expanding from core stations to approximately twice the interval, accompanied by an increased propagation rate. Additionally, the propagation duration is significantly extended, shifting from rapid attenuation in L1 to sustained effects even after the peak period under L3. Overall, lower degraded operations result in stronger, bRd.er, and longer-lasting passenger flow propagation, following a stepwise diffusion pattern from core to adjacent and downstream stations. From a network-level perspective, the accumulated passenger demand and excess queuing pressure caused by reduced capacity in a local section propagate outward along train operating directions and passenger alternative routes. This transmission leads to simultaneous increases in inbound passenger flow and waiting passengers at adjacent and downstream stations, resulting in a broader spatial overlap between rising passenger density and insufficient capacity.

**Fig 16:**
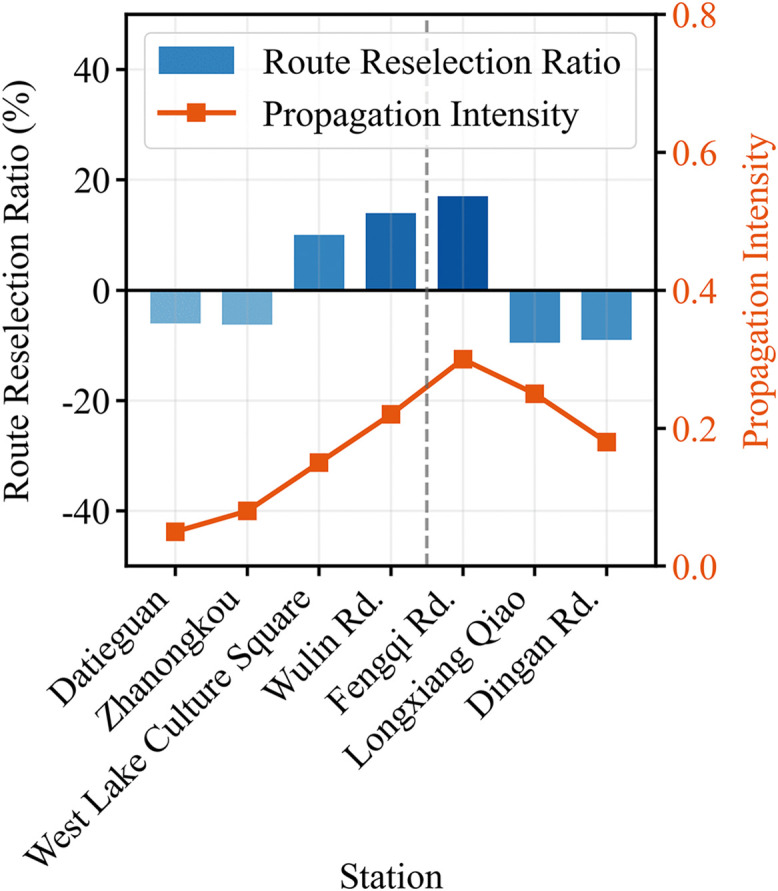
Impact of Passenger Route Reselection on Flow Propagation Intensity Under L1 Degraded Operations.

**Fig 17:**
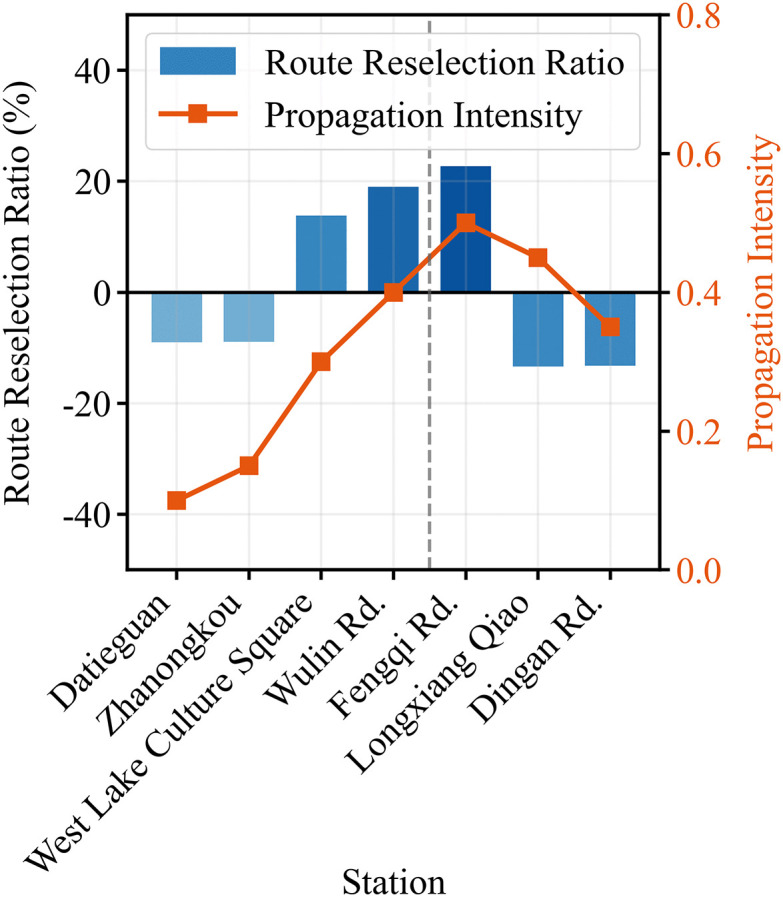
Impact of Passenger Route Reselection on Flow Propagation Intensity Under L2 Degraded Operations.

**Fig 18:**
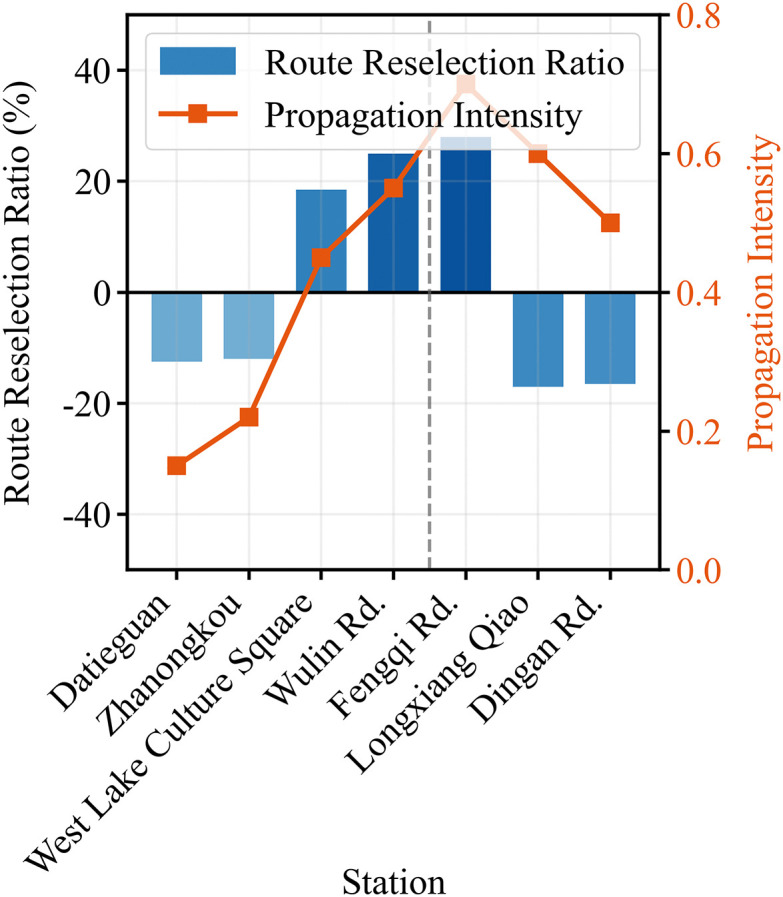
Impact of Passenger Route Reselection on Flow Propagation Intensity Under L3 Degraded Operations.

To further investigate the moderating effect of passenger path re-selection behavior on propagation intensity under different degraded operations, Figs 16–18 presents the changes in propagation intensity from L1 to L3 when path re-selection is considered.

As shown in Figs 16–18, the route re-selection ratio and passenger flow propagation intensity show distinct changes as the degraded operations de-escalates from L1 to L3. In L1 mode, the re-selection ratio is approximately 10%–17%, with propagation intensity around 0.3. As the mode transitions to L2, the re-selection ratio increases to around 20%, and propagation intensity rises to 0.5, particularly at stations like Wulin Rd. and Longxiang Qiao, where intensity significantly amplifies. In L3 mode, the re-selection ratio further increases to nearly 30%, and propagation intensity generally rises to 0.7, peaking at Fengqi Rd. where the re-selection ratio reaches nearly 30%, and propagation intensity is at its highest. These results indicate that path re-selection significantly accelerates the spread of local pressure and facilitates the formation of propagation chains under different degraded operations.

### 5.5. Sensitivity analysis of bounded rationality threshold parameters

To examine the responsiveness of the proposed bounded rationality route choice model to key threshold parameters and to verify its structural stability within behaviorally plausible ranges, a systematic sensitivity analysis was conducted for the rationality coefficient (*μ*_*m*_), the waiting time tolerance threshold ($\overset{m}{\underset{1}{\gamma }}$), and the degradation tolerance threshold ($\overset{m}{\underset{2}{\gamma }}$). The analysis focuses on route reselection behavior, evaluated using the average reselection rate (R) and the choice concentration index (HHI).

(1) Sensitivity Analysis of the Rationality Coefficient (*μ*_*m*_)

The rationality coefficient *μ*_*m*_ determines the dispersion level of route choice probabilities. In this study, was tested over an extended interval *μ*_*m*_ ∈ [2, 40], with the calibrated range defined as *μ*_*m*_ ∈ [14, 16]. The numerical results corresponding to different *μ*_*m*_ values are presented in Table 8.

**Table 8:** Sensitivity results for different values of *μ*_*m*_.

*μ*_*m*_	Average Reselection Rate (R)	Choice Concentration Index (HHI)
2	0.095	0.18
5	0.140	0.26
8	0.170	0.33
12	0.205	0.38
14	0.230	0.42
16	0.247	0.44
18	0.258	0.46
20	0.270	0.48
25	0.295	0.55
30	0.340	0.68
40	0.395	0.81

**Fig 19:**
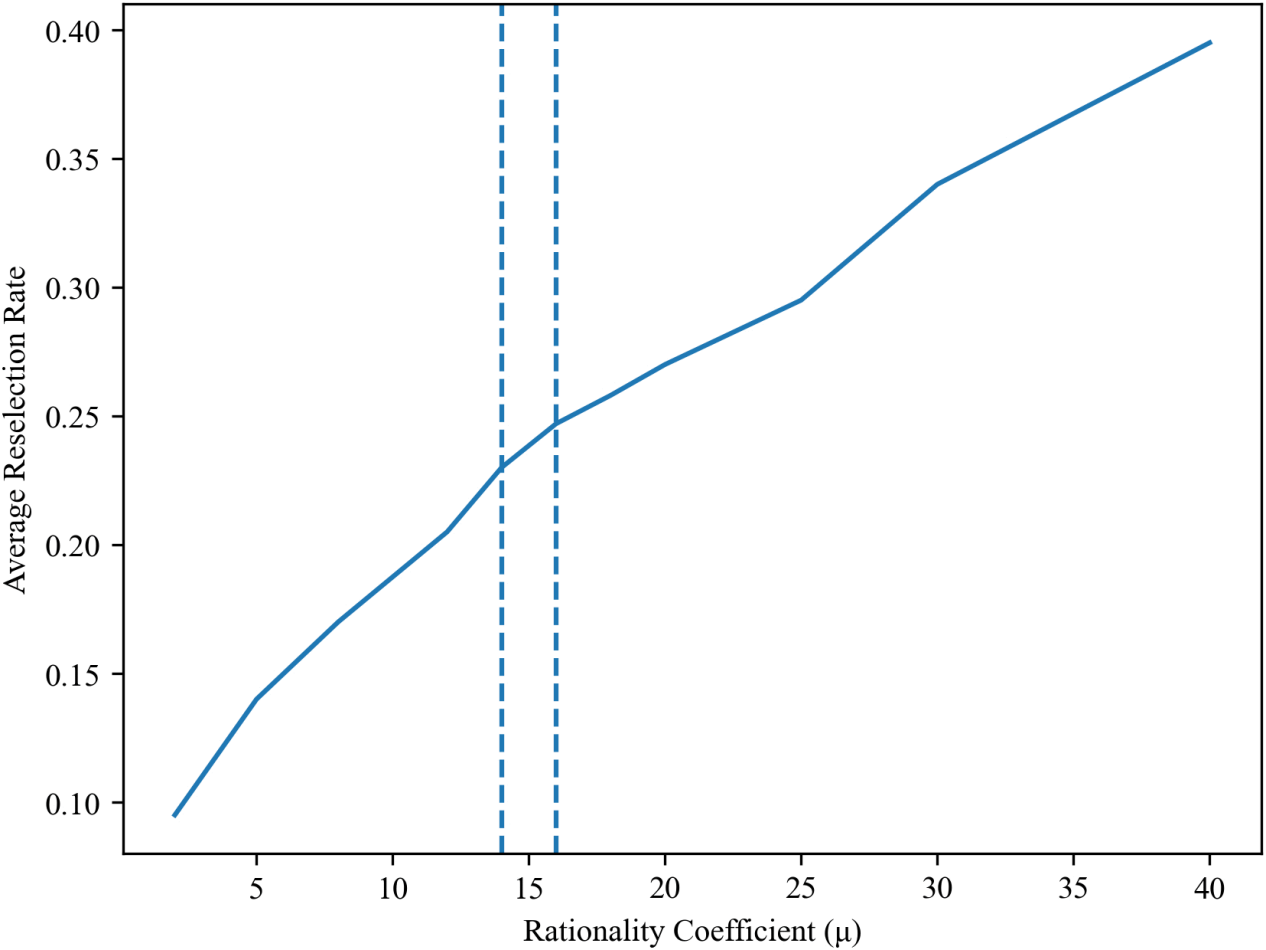
Sensitivity Analysis of Route Reselection Rate With Respect to the Rationality Coefficient (*μ*_*m*_).

**Fig 20:**
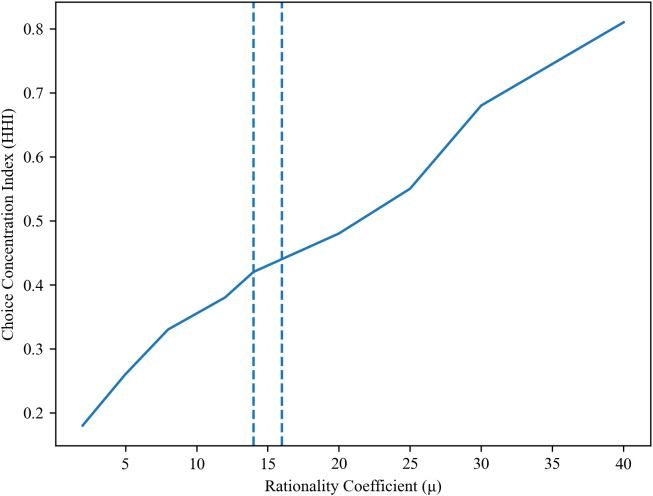
Sensitivity Analysis of Choice Concentration Index (HHI) With Respect to the Rationality Coefficient (*μ*_*m*_).

Table 8 summarizes the quantitative impact of *μ*_*m*_ on the scale of route reselection and the concentration of route choice. Based on these results, Figs 19-20 depict the functional relationships between *μ*_*m*_ and R, and between *μ*_*m*_ and HHI, respectively.

As shown in Table 8 and Figs 19–20, both the average reselection rate and the choice concentration index increase monotonically with *μ*_*m*_. Within the calibrated interval *μ*_*m*_ ∈ [14,16], the variation in R is moderate and smooth, while HHI exhibits a gradual upward trend without any structural break or abrupt transition. When *μ*_*m*_ exceeds approximately 25, the growth rate of HHI accelerates significantly, indicating that route choice becomes increasingly concentrated. Conversely, when *μ*_*m*_ is below 8, the concentration level decreases markedly, reflecting more stochastic route choice behavior. These results indicate that the model remains structurally stable within the calibrated interval, whereas extreme rationality levels may induce nonlinear amplification effects.

(2) Sensitivity Analysis of the Waiting Time Tolerance Threshold ($\overset{m}{\underset{1}{\gamma }}$)

The waiting time tolerance threshold $\overset{m}{\underset{1}{\gamma }}$ represents passengers’ tolerance for deviations from their reference waiting time. The parameter was tested over an extended interval $\overset{m}{\underset{1}{\gamma }}$ ∈ [0.2, 1.2], with the calibrated range defined as $\overset{m}{\underset{1}{\gamma }}$ ∈ [0.6, 0.8]. The corresponding reselection rates are reported in Table 8.

**Fig 21:**
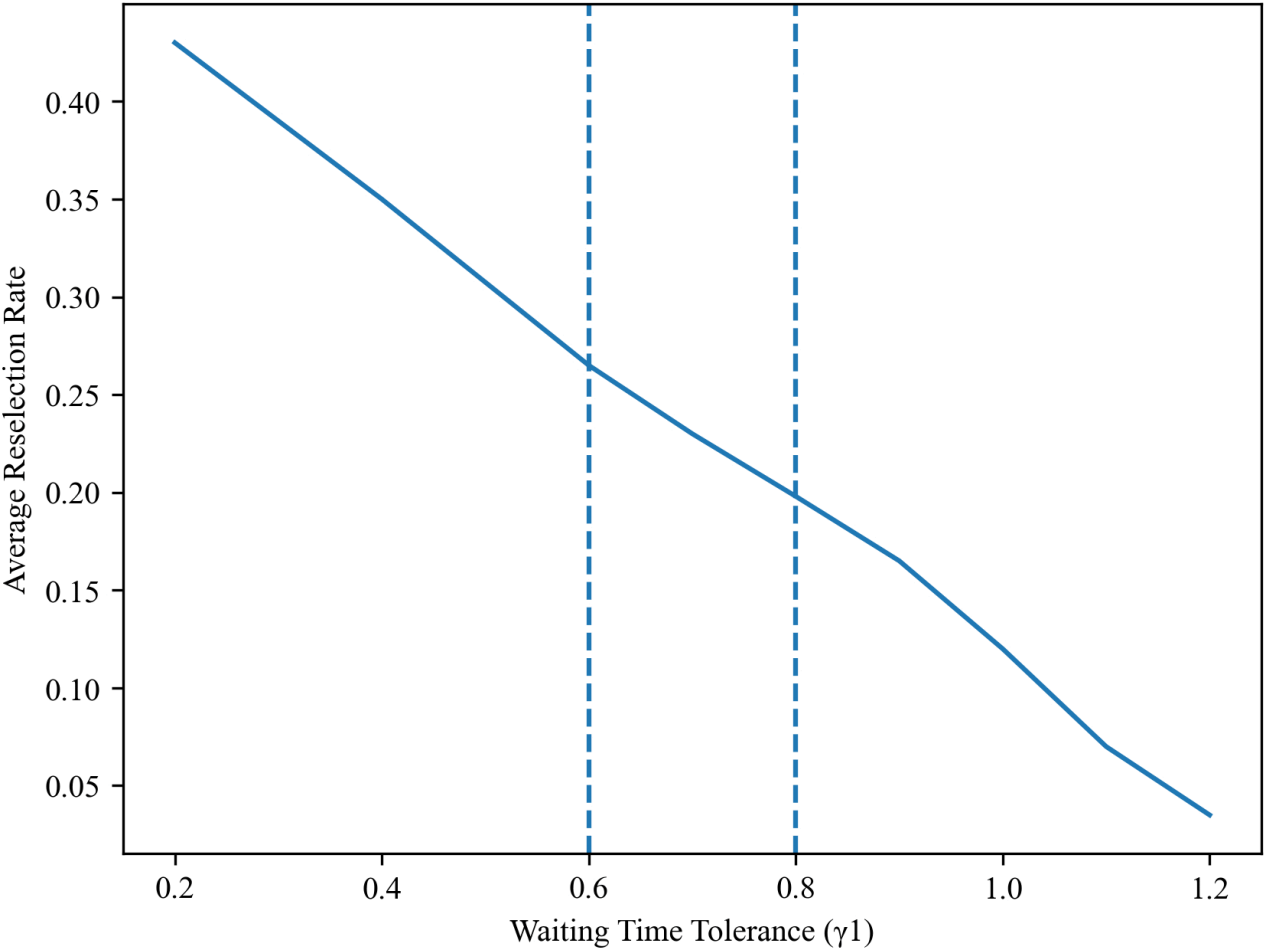
Sensitivity analysis of route reselection behavior with respect to the waiting time tolerance ($\overset{m}{\underset{1}{\gamma }}$).

Table 9 provides the numerical impact of $\overset{m}{\underset{1}{\gamma }}$ on the route reselection rate. Based on these data, Fig 21 illustrates the variation of R with respect to $\overset{m}{\underset{1}{\gamma }}$.

**Table 9:** Sensitivity results for different values of $\overset{m}{\underset{1}{\gamma }}$.

$\overset{m}{\underset{1}{\gamma }}$	0.2	0.4	0.6	0.7	0.8	0.9	1.0	1.1	1.2
**R**	0.430	0.350	0.265	0.230	0.198	0.165	0.120	0.070	0.035

As observed from Table 9 and Fig 21, the reselection rate decreases monotonically as $\overset{m}{\underset{1}{\gamma }}$ increases. Within the calibrated interval, the variation in R is smooth and continuous, with no abrupt change or non-monotonic pattern. When $\overset{m}{\underset{1}{\gamma }}$ is below 0.4, reselection behavior increases substantially; when $\overset{m}{\underset{1}{\gamma }}$ exceeds 1.0, the reselection rate declines sharply, indicating a significant weakening of the triggering mechanism. These findings suggest that the model exhibits stable responsiveness to moderate variations in $\overset{m}{\underset{1}{\gamma }}$ within the calibrated range.

(3) Sensitivity Analysis of the Degradation Tolerance Threshold ($\overset{m}{\underset{2}{\gamma }}$)

The degradation tolerance threshold $\overset{m}{\underset{2}{\gamma }}$ reflects passengers’ tolerance toward perceived service deterioration. The parameter was tested over $\overset{m}{\underset{2}{\gamma }}$ ∈ [0.1, 1.0], with the calibrated interval defined as $\overset{m}{\underset{2}{\gamma }}$ ∈ [0.4, 0.6]. The resulting reselection rates are shown in Table 10.

**Table 10:** Sensitivity results for different values of.

$\overset{m}{\underset{2}{\gamma }}$	0.1	0.2	0.3	0.4	0.5	0.6	0.7	0.8	0.9	1.0
**R**	0.390	0.330	0.275	0.245	0.230	0.215	0.190	0.160	0.115	0.070

**Fig 22:**
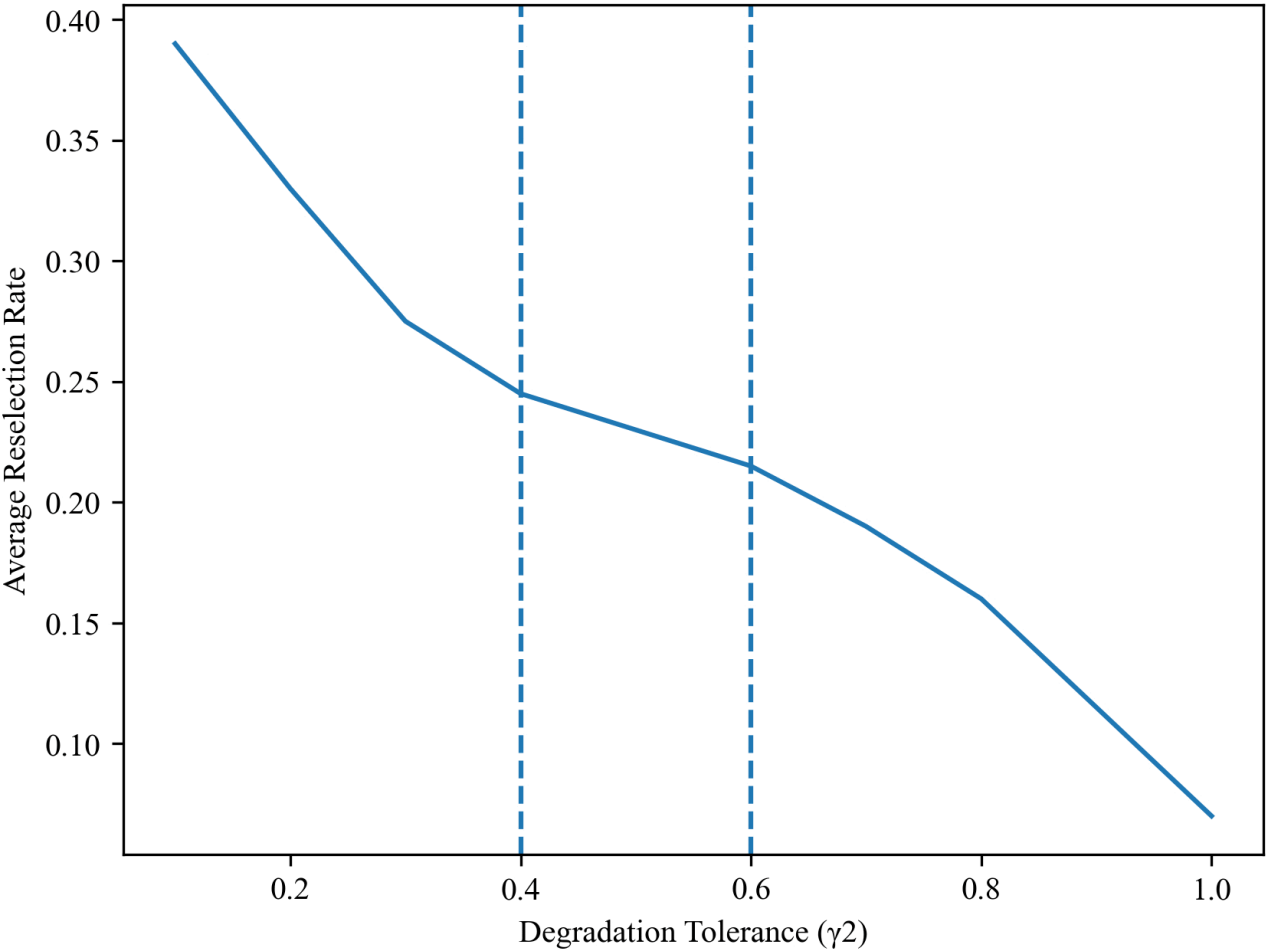
Sensitivity analysis of route reselection behavior with respect to the degradation tolerance ($\overset{m}{\underset{2}{\gamma }}$).

Table 9 presents the quantitative effect of $\overset{m}{\underset{2}{\gamma }}$ on the route reselection rate. Fig 22 further visualizes the relationship between $\overset{m}{\underset{2}{\gamma }}$ and R.

As shown in Table 10 and Fig 22, the reselection rate decreases monotonically as $\overset{m}{\underset{2}{\gamma }}$ increases. Within the calibrated interval, the variation in R remains smooth and stable. When $\overset{m}{\underset{2}{\gamma }}$ takes extreme values, the behavioral response exhibits noticeable amplification or suppression effects.

A comprehensive comparison of Tables 8–10 and Figs 19–22 indicates that, within the calibrated intervals adopted in this study, the effects of bounded rationality threshold parameters on route reselection behavior are continuous and smooth, without structural breaks or non-monotonic transitions. Although nonlinear responses emerge when parameters deviate substantially from behaviorally plausible ranges, the overall behavioral patterns remain consistent.

## 6. Conclusion

This study investigates passenger-train coordination characteristics and passenger flow evolution mechanisms in urban rail transit under degraded operations. An integrated analytical framework incorporates path generation and identification, bounded rationality route choice behavior, and passenger flow propagation processes. The modeling approach integrates degradation utility functions, route familiarity factors, and dynamic rationality coefficients to scientifically characterize the coupling relationship between passenger behavioral adaptations and system operational states.

The research results indicate that under degraded operation conditions, the model incorporating bounded rationality assumptions is more consistent with actual data. In the L2 degradation scenario, the deviation between the bounded rationality model and the observed values is significantly smaller than that of the fully rational model. This finding suggests that under service capacity constraints, introducing bounded rationality assumptions improves the empirical fit of route reselection modeling.

The passenger-train coordinated dynamic route simulation model successfully visualizes the complete evolution pattern of “core accumulation - adjacent diffusion - regional retention” under degraded operations. Simulation results demonstrate clear trends of increasing passenger flow saturation at core stations and enhanced propagation intensity across network sections as operational conditions worsen. The model quantitatively characterizes interrelationships among passenger flow saturation levels, propagation scope expansion, duration extension, and passenger flow intensity pressure trends.

Although this study does not directly verify the cognitive mechanisms underlying passengers’ bounded rational decision-making, the comparative results demonstrate that under degraded operation scenarios, the model incorporating bounded rationality assumptions is closer to actual passenger behavior than the fully rational benchmark model. This indicates that when service capacity declines, the bounded rationality assumption contributes to a more realistic representation of passenger response characteristics.

## 7. Discussion

To incorporate behavioral heterogeneity in a computationally tractable manner, this study adopts a structured parameter stratification strategy. Time-sensitive passengers exhibit high sensitivity to time loss and service disruptions; route-dependent passengers demonstrate stable preferences for familiar routes; and adaptive-resilient passengers show greater tolerance toward waiting and delays. Passenger types are assigned upon system entry and remain unchanged throughout the entire travel process. All individual-level parameters are strictly constrained within the intervals specified in Table 4. To introduce structured heterogeneity, each parameter interval [a,b] is evenly divided into three sub-intervals: the lower third [a,a+b-a/3], the middle third [a+b-a/3,a+2(b-a)/3], and the upper third [a+2(b-a)/3,b], with the three passenger types assigned values from different sub-intervals accordingly.

Specifically, in the time valuation dimension, the psychological reference time *t_ref_* ∈[1.0,1.5], time sensitivity coefficient *η_t_* ∈[1.2,1.8], delay sensitivity coefficient *ξ_t_* ∈[0.3,1], and delay aversion coefficient Γ*_t_* ∈[1.5,2.5] are assigned from the upper-third interval for time-sensitive passengers, the middle-third interval for route-dependent passengers, and the lower-third interval for adaptive-resilient passengers. In the transfer and congestion dimension, the transfer penalty coefficient *λ_H_* ∈[0,2] and congestion sensitivity parameter *ζ_t_* ∈[0.1,0.8] follow the same stratification rule. For degradation-related parameters, the degradation sensitivity coefficient *λ_L_* ∈[0,1] is assigned from the upper-third interval for time-sensitive passengers, while the degradation tolerance coefficient $\overset{m}{\underset{2}{\gamma }}$ ∈[0.4,0.6] follows a reverse distribution pattern, with time-sensitive passengers assigned to the lower-third interval and adaptive-resilient passengers to the upper-third interval. The waiting time tolerance coefficient $\overset{m}{\underset{1}{\gamma }}$ ∈[0.6,0.8] increases progressively from time-sensitive to adaptive-resilient passengers. For familiarity-related parameters, the familiarity utility gain coefficient *λ*_*F*_ ∈[0,1] and route familiarity adjustment coefficient *ξ*_*L*_ ∈[0.5,0.7] are assigned from the upper-third interval for route-dependent passengers, with the remaining types allocated progressively lower ranges. The rationality coefficient *μ*_*m*_ ∈ [14,16] is hierarchically initialized, with route-dependent passengers assigned to the upper interval, time-sensitive passengers to the middle interval, and adaptive-resilient passengers to the lower interval.

During parameter assignment, passenger type labels are first allocated according to predefined proportions. For each parameter, specific values are then generated through uniform random sampling within the corresponding sub-interval of the assigned passenger type. All parameters remain fixed throughout the travel process, except for the rationality coefficient *μ*_*m*_, which dynamically adjusts with waiting time and degradation level. Through the three-step mechanism of “interval partitioning – type matching – within-interval random generation,” the model establishes a heterogeneity structure that simultaneously captures categorical behavioral differences and preserves individual stochastic variability, while ensuring that all parameter values remain strictly bounded within the ranges specified in Table 4.

In the simulation, behavioral heterogeneity is implemented at the individual passenger level. For each passenger *m* entering the system, a categorical indicator $z_{m}$ ∈{1,2,3} is first generated. The assignment follows a discrete distribution with weights (π_1_,π_2_,π_3_), satisfying π_1_ + π_2_ + π_3_ = 1. In the baseline configuration, equal weights π_1_ = π_2_ = π_3_ = are adopted to avoid structural bias toward any specific behavioral range.

Once $z_{m}$ is determined, the corresponding parameter sub-interval is activated for each behavioral parameter. Let the calibrated interval of a parameter be [a,b]. This interval is evenly partitioned into three sub-intervals:

$\left[a,a+\frac{b-a}{3}\right],\;\left[a+\frac{b-a}{3},a+\frac{2\left(b-a\right)}{3}\right],[a+\frac{2(b-a)}{3},b].$

If $z_{m}$ =*k*, the parameter value for passenger *m* can be generated by uniform random sampling within the *k*-th sub-interval.

This procedure is consistently applied to all time-related, transfer-related, congestion-related, degradation-related, and familiarity-related parameters. All parameters remain fixed during the passenger’s trip except for the rationality coefficient, which dynamically evolves according to Equation (18) as a function of waiting time and degradation intensity.

Through this assignment and generation mechanism, all parameter sub-intervals can be represented within the simulated passenger population, while avoiding structural bias caused by concentrating individuals around a single representative value. The combination of probabilistic allocation and interval-based parameter generation can introduce individual-level heterogeneity while preserving consistency with the calibrated parameter ranges. This design allows the model to reflect differentiated behavioral sensitivities while maintaining numerical stability and computational tractability.

## 8. Futurework

Building upon this study’s investigation into passenger accumulation formation mechanisms, future research will prioritize examining how passenger flow control measures influence the dissipation processes of congestion and accumulation. While the current research has elucidated the “core accumulation - adjacent diffusion - regional retention” evolution pattern, the operational mechanisms through which various control measures accelerate accumulation dissipation remain inadequately understood and will constitute a central research focus.

Specific research directions will include: (1) developing quantitative models correlating control measures with dissipation efficiency, particularly analyzing how critical interventions—including station-level flow restrictions, train scheduling optimization, and route guidance strategies—affect accumulation dissipation rates; (2) investigating synergistic mechanisms of multiple control measures to determine optimal spatiotemporal configuration schemes for different control combinations; and (3) creating dynamic control systems that utilize real-time passenger flow data to achieve adaptive alignment between control intensity and accumulation levels.

This research aims to establish a comprehensive theoretical framework for the complete “accumulation-dissipation” process, with particular emphasis on deciphering the mechanistic role of control measures during the dissipation phase. By systematically evaluating how control measures regulate dissipation processes across diverse scenarios, we ultimately seek to develop an intelligent decision-support system for passenger flow management in urban rail transit networks.
