# Correction: Research on passenger-train coordination and passenger flow evolution in Urban rail transit systems under degraded operations

**DOI:** 10.1371/journal.pone.0355407

**Published:** 2026-08-04

**Authors:** Jing Zuo, Minggan Xue, Yuanxian Zhao, Junting Lin

Minggan Xue is not included in the author byline. Minggan Xue should be listed as the second author and affiliated with School of Automation and Electrical Engineering, Lanzhou Jiaotong University, Lanzhou, China. The contributions of this author are as follows: Conceptualization, Data Curation, Formal Analysis, Investigation, Methodology, Software, Supervision, Validation, Visualization, Writing – Original Draft and Writing – Review & Editing.

The correct citation is: Zuo J, Xue M, Zhao Y, Lin J (2026) Research on passenger-train coordination and passenger flow evolution in Urban rail transit systems under degraded operations. PLoS One 21(7): e0349898. https://doi.org/10.1371/journal.pone.0349898
