# Supplementary material for: Research on passenger-train coordination and passenger flow evolution in Urban rail transit systems under degraded operations
Source: PLoS One. 2026 Jul 6;21(7):e0349898. doi: 10.1371/journal.pone.0349898 (PMC13336208; doi:10.1371/journal.pone.0349898)
Supplement: Attachment — Submitted filename: Response to Reviewers.docx [file pone.0349898.s002.docx]

**Response to the Reviewers’ Comments on the Manuscript
“Research on Passenger-Train Coordination and Passenger Flow Evolution in Urban Rail Transit Systems under Degraded Operations”**

Dear Editor and Reviewers,

We sincerely appreciate the time and effort you have devoted to reviewing our manuscript entitled “Research on Passenger-Train Coordination and Passenger Flow Evolution in Urban Rail Transit Systems under Degraded Operations.” We are deeply grateful for your valuable comments and constructive suggestions. Your careful evaluation and professional guidance have significantly helped us improve the clarity, scientific rigor, and overall quality of the manuscript.

We have carefully considered all the comments and suggestions provided by the Editor and Reviewers, and have made corresponding revisions and improvements throughout the manuscript. We hope that the revised version meets the standards and expectations of the journal.

The authors have addressed each comment point-by-point in the revised manuscript. Below, we provide detailed responses to the reviewers’ comments：

Reviewer #1

1. The paper concludes that "passenger route choice under degraded operations exhibits bounded rationality," which is not sufficiently supported by the presented results. The bounded rationality assumption is introduced at the modeling stage, and the subsequent calibration and simulation validate the performance of the model rather than the bounded rationality hypothesis itself.

**Response：**

We sincerely thank the reviewer for pointing out the logical ambiguity in our original statement regarding the conclusion that “passenger route choice exhibits bounded rationality characteristics.” Upon careful reconsideration, we acknowledge that the previous wording may have been interpreted as a direct empirical validation of passengers’ cognitive mechanisms. However, the primary objective of this study is to compare the degree of consistency between different decision-making assumptions and observed data, rather than to empirically verify the psychological mechanism of bounded rationality itself.

In response to the reviewer’s valuable suggestion, we have systematically revised the manuscript as follows:

(1) In the Abstract, Results, and Conclusion sections, we have removed statements such as “passengers exhibit bounded rationality characteristics” that could imply a direct validation of cognitive mechanisms. These expressions have been reformulated within a model comparison framework, explicitly clarifying that our conclusions are derived from comparative analyses between the bounded rationality model and a fully rational benchmark model.

(2) In terms of data presentation, we have supplemented the results of the fully rational model under the three degradation scenarios (L1, L2, and L3), and added the corresponding observed data under the L2 degradation scenario. By presenting the bounded rationality model, the fully rational model, and the observed data in parallel, the comparative consistency of the models becomes more transparent and intuitive.

(3) In the Results section, we now emphasize that under the L2 degradation scenario, the bounded rationality model demonstrates closer agreement with the observed data than the fully rational model. This finding indicates that incorporating bounded rationality assumptions enhances the empirical consistency of the model.

(4) In the Conclusion section, we have added clarifying statements to explicitly indicate that this study does not aim to directly validate the cognitive mechanism of bounded rationality. Instead, the analysis is conducted from the perspective of model explanatory power and empirical fit.

**The specific revisions are detailed below：**

①**Original version**：Simulation experiments using Hangzhou Metro data demonstrate that passenger route choice exhibits bounded rationality characteristics, with reselection rates increasing from 12% to 38% as service level degrades from L1 to L3.

**Revised version**：The experimental results indicate that under abnormal train operation scenarios, the model incorporating bounded rationality assumptions is closer to actual observed values compared with the fully rational benchmark model. In the L2 degradation scenario, the deviation between the bounded rationality model and the actual values is controlled within 0.6%–1.3%, whereas the deviation of the fully rational model is approximately 12%.

②**Original version**：**Table 6. Peak Proportion of Passenger Path Re-selection in the Interlocking Area under Different Degraded operations**

| **Station** | **L1** | **L2** | **L3** |
| --- | --- | --- | --- |
| West Lake Cultural Square | 12.5% | 18.9% | 24.3% |
| Wulin Rd. | 15.8% | 23.6% | 31.6% |
| Fengqi Rd. | 18.3% | 28.1% | 38.2% |

Table 6 shows that as the severity of degraded operations increases, the peak path re-selection rate at stations within the interlocking area progressively rises. Specifically, the peak re-selection rate at Fengqi Rd. Station increases from 18.3% under L1 to 28.1% under L2, and further to 38.2% under L3, while Wulin Rd. Station and West Lake Cultural Square Station exhibit similar trends. This highlights the significant impact of degraded operations changes on passenger path choices, with passengers more likely to switch routes as degraded operations intensify.

**Revised version**：**Table 6. Peak Proportion of Passenger Path Re-selection in the Interlocking Area under Different Degraded operations**

| **Station** | **L1 (Bounded Rationality)** | **L1 (Fully Rational)** | **L2 (Actual Value)** | **L2 (Bounded Rationality)** | **L2 (Fully Rational)** | **L3 (Bounded Rationality)** | **L3 (Fully Rational)** |
| --- | --- | --- | --- | --- | --- | --- | --- |
| West Lake Cultural Square | 12.5% | 21.4% | 19.8% | 18.9% | 32.7% | 24.3% | 45.8% |
| Wulin Rd. | 15.8% | 24.6% | 24.2% | 23.6% | 36.9% | 31.6% | 50.2% |
| Fengqi Rd. | 18.3% | 27.3% | 29.4% | 28.1% | 41.5% | 38.2% | 56.4% |

Table 6 shows that as the severity of degraded operations increases, the peak path re-selection rate at stations within the interlocking area progressively rises. Specifically, the peak re-selection rate at Fengqi Rd. Station increases from 18.3% under L1 to 28.1% under L2, and further to 38.2% under L3, while Wulin Rd. Station and West Lake Cultural Square Station exhibit similar trends. This highlights the significant impact of degraded operations changes on passenger path choices, with passengers more likely to switch routes as degraded operations intensify.

To compare the consistency between model results under different rationality assumptions and actual observations, passenger route reselection rates under the fully rational and bounded rationality settings were first compared. On this basis, the observed values under the L2 degraded operation scenario were selected as the reference benchmark for further analysis. The results indicate that under all degraded operation levels, the fully rational model yields higher route reselection proportions than the bounded rationality model, and the gap between the two models widens as the degradation level increases. The difference intervals between the two model results under L1, L2, and L3 are [8.8%, 9.0%], [13.3%, 13.8%], and [18.2%, 21.5%], respectively.

Under the L2 degraded scenario, the results of both rationality assumptions were further compared with the actual observed values. At West Lake Cultural Square Station, the deviations of the bounded rationality and fully rational models are 0.9% and 12.9%, respectively; at Wulin Rd. Station, they are 0.6% and 12.7%; and at Fengqi Rd. Station, they are 1.3% and 12.1%. Correspondingly, the deviation range of the bounded rationality model is 0.6%–1.3%, whereas that of the fully rational model is 12.1%–12.9%. A clear disparity in deviation magnitude is observed between the two models, with the bounded rationality model exhibiting substantially smaller numerical deviations from the actual observations overall.

③**Original version**：This study investigates passenger-train coordination characteristics and passenger flow evolution mechanisms in urban rail transit under degraded operations. An integrated analytical framework incorporates path generation and identification, bounded rationality route choice behavior, and passenger flow propagation processes. The modeling approach integrates degradation utility functions, route familiarity factors, and dynamic rationality coefficients to scientifically characterize the coupling relationship between passenger behavioral adaptations and system operational states.

Research findings demonstrate that passenger route selection under degraded operations exhibits bounded rationality with distinct perceptual biases and cognitive constraints. Simulation experiments reveal significantly increased route reselection rates as service levels deteriorate. Passenger decision-making shows simultaneous influence from multiple factors including psychological time perception, route familiarity, and crowding sensitivity, collectively triggering alternative route selection behavior. The bounded rationality model effectively captures systematic deviations between subjective perceptions and objectively optimal choices during service degradation.

The passenger-train coordinated dynamic route simulation model successfully visualizes the complete evolution pattern of "core accumulation - adjacent diffusion - regional retention" under degraded operations. Simulation results demonstrate clear trends of increasing passenger flow saturation at core stations and enhanced propagation intensity across network sections as operational conditions worsen. The model quantitatively characterizes interrelationships among passenger flow saturation levels, propagation scope expansion, duration extension, and passenger flow intensity pressure trends.

**Revised version**：

This study investigates passenger-train coordination characteristics and passenger flow evolution mechanisms in urban rail transit under degraded operations. An integrated analytical framework incorporates path generation and identification, bounded rationality route choice behavior, and passenger flow propagation processes. The modeling approach integrates degradation utility functions, route familiarity factors, and dynamic rationality coefficients to scientifically characterize the coupling relationship between passenger behavioral adaptations and system operational states.

The research results indicate that under degraded operation conditions, the model incorporating bounded rationality assumptions is more consistent with actual data. In the L2 degradation scenario, the deviation between the bounded rationality model and the observed values is significantly smaller than that of the fully rational model. This finding suggests that under service capacity constraints, introducing bounded rationality assumptions improves the empirical fit of route reselection modeling.

The passenger-train coordinated dynamic route simulation model successfully visualizes the complete evolution pattern of "core accumulation - adjacent diffusion - regional retention" under degraded operations. Simulation results demonstrate clear trends of increasing passenger flow saturation at core stations and enhanced propagation intensity across network sections as operational conditions worsen. The model quantitatively characterizes interrelationships among passenger flow saturation levels, propagation scope expansion, duration extension, and passenger flow intensity pressure trends.

Although this study does not directly verify the cognitive mechanisms underlying passengers’ bounded rational decision-making, the comparative results demonstrate that under degraded operation scenarios, the model incorporating bounded rationality assumptions is closer to actual passenger behavior than the fully rational benchmark model. This indicates that when service capacity declines, the bounded rationality assumption contributes to a more realistic representation of passenger response characteristics.

2. The paper assumes bounded rationality in passenger route choice and calibrates several behavioral parameters; however, the calibration process and data sources for many key parameters are not clearly explained.

**Response：**

We sincerely thank the reviewer for the valuable comment. Upon careful re-examination of the original manuscript, we acknowledge that the calibration process and data sources of several key behavioral parameters were not described in sufficient detail, and that some related explanations lacked clarity.

In response to this concern, we have expanded the description of the parameter-setting procedures in the revised manuscript. Specifically, we systematically clarified the sources and estimation methods for different categories of parameters. Operational time-related parameters are calibrated in a data-driven manner based on AFC records and train timetables. Behavioral perception parameters are constrained with reference to established empirical studies while being adapted to the proposed modeling framework. Key decision-making parameters are calibrated using a likelihood-based approach grounded in observed path distribution data, and the corresponding optimization objectives and estimation procedures are now explicitly described. These revisions enhance the transparency and reproducibility of the parameter-setting process.

**The revised text is provided as follows:**

**Original version**：The parameters of the bounded rational path re-selection model are determined within a range based on passenger surveys, AFC data analysis, and related literature, as presented in Table 4.

**Revised version**：During the parameterization of the bounded rational route reselection model, this study adopts a systematic calibration framework that integrates data-driven analysis with evidence from the existing literature. This approach ensures that the model is both empirically grounded and theoretically consistent with established behavioral theories. The parameters are categorized into three groups: operational time-related parameters, behavioral perception parameters, and key decision-making parameters. Each group is estimated using a targeted calibration strategy.

First, parameters related to path identification and operational travel time are jointly estimated using AFC data and train timetables. By matching observed travel times from AFC records with theoretically calculated travel times derived from timetables, the distribution of time deviations is obtained. An optimization model is then constructed with the objective of minimizing the Mean Squared Error (MSE) between observed and estimated travel times, and the walking time coefficient is calibrated accordingly. Through statistical optimization, the walking time coefficient is determined such that the model-estimated travel times are statistically consistent with observed values, thereby improving the accuracy of path identification and the reliability of travel time representation.

Second, behavioral perception parameters—including the time sensitivity coefficient, delay aversion coefficient, and rationality coefficient—are primarily determined based on empirically validated parameter ranges reported in prior studies on rail transit route choice behavior and prospect theory-related research [27–33]. These parameter ranges are further constrained to ensure compatibility with the bounded rational decision-making framework developed in this study. Such parameters characterize passengers’ psychological perception intensity under delay conditions, risk-averse tendencies, and bounded rationality attributes. The adopted intervals ensure consistency with established empirical findings while preventing parameter values that would deviate from recognized behavioral regularities.

Finally, key behavioral parameters that directly influence the probability distribution of route choice—such as the transfer penalty coefficient and the degradation sensitivity coefficient—are calibrated using a likelihood-based estimation approach grounded in observed data. Specifically, the actual route choice distribution is constructed based on AFC-based path identification results. The route choice probabilities generated by the model are then compared with the observed distribution, and the log-likelihood function is employed as the goodness-of-fit metric. A discrete parameter search is conducted within predefined feasible intervals, and the parameter combination that maximizes the log-likelihood value is selected as the final estimate, thereby completing the calibration of the key behavioral parameters.

Based on the above statistical analysis and optimization procedures, the calibrated parameter ranges of the model are summarized in Table 4.

3. Many of the behavioral parameters in the model are presented only as ranges, and the paper does not specify how these ranges are mapped to individual passengers (such as parameter distributions like normal, lognormal, or empirical distributions). Also, the model explicitly incorporates passenger heterogeneity, while it is unclear in the model and formula.

**Response：**

We sincerely thank the reviewer for this important comment. We acknowledge that the original manuscript did not provide sufficient clarification regarding the implementation of passenger heterogeneity, which may have affected the clarity and reproducibility of the model structure.

In the revised manuscript, we have added a dedicated subsection in the Discussion section to systematically explain the passenger classification approach and the parameter assignment mechanism. Specifically, passengers are categorized into three behavioral types: time-sensitive, path-dependent, and adaptive. All individual-level parameters are strictly constrained within the calibrated intervals reported in Table 4.

To introduce structural heterogeneity without altering the original parameter calibration framework, each parameter interval is evenly divided into three sub-intervals. Parameter values for time-sensitive passengers are drawn from the upper sub-interval, values for adaptive passengers are drawn from the lower sub-interval, and values for path-dependent passengers are drawn from the middle sub-interval.

For tolerance-related parameters (e.g., waiting time tolerance), an inverse gradient assignment is applied to maintain behavioral consistency. Specific parameter values are randomly generated within the corresponding sub-interval and remain fixed throughout an individual’s entire trip. The only parameter that varies dynamically is the bounded rationality coefficient, which adjusts in response to disturbance conditions.

Through these revisions, we ensure that:
(1) All parameters strictly adhere to the calibrated intervals reported in Table 4;
(2) Passenger heterogeneity is explicitly implemented at the individual level;
(3) Structural behavioral differences are preserved without introducing additional free parameters;
(4) The model implementation process becomes more transparent, thereby enhancing reproducibility.

We believe that these additions substantially improve the clarity and methodological rigor of the model specification.

**The detailed revisions are provided as follows:**

To incorporate behavioral heterogeneity in a computationally tractable manner, this study adopts a structured parameter stratification strategy. Time-sensitive passengers exhibit high sensitivity to time loss and service disruptions; route-dependent passengers demonstrate stable preferences for familiar routes; and adaptive-resilient passengers show greater tolerance toward waiting and delays. Passenger types are assigned upon system entry and remain unchanged throughout the entire travel process. All individual-level parameters are strictly constrained within the intervals specified in Table 4. To introduce structured heterogeneity, each parameter interval [a,b] is evenly divided into three sub-intervals: the lower third [a,a+], the middle third [a+,a+], and the upper third [a+,b], with the three passenger types assigned values from different sub-intervals accordingly.

Specifically, in the time valuation dimension, the psychological reference time $t_{ref}$∈[1.0,1.5], time sensitivity coefficient $\eta_{t}$∈[1.2,1.8], delay sensitivity coefficient $\xi_{t}$∈[0.3,1], and delay aversion coefficient $\Gamma_{t}$∈[1.5,2.5] are assigned from the upper-third interval for time-sensitive passengers, the middle-third interval for route-dependent passengers, and the lower-third interval for adaptive-resilient passengers. In the transfer and congestion dimension, the transfer penalty coefficient $\lambda_{H}$∈[0,2] and congestion sensitivity parameter $\zeta_{H}$∈[0.1,0.8] follow the same stratification rule. For degradation-related parameters, the degradation sensitivity coefficient $\lambda_{L}$∈[0,1] is assigned from the upper-third interval for time-sensitive passengers, while the degradation tolerance coefficient $\gamma_{2}^{m}$∈[0.4,0.6] follows a reverse distribution pattern, with time-sensitive passengers assigned to the lower-third interval and adaptive-resilient passengers to the upper-third interval. The waiting time tolerance coefficient $\gamma_{1}^{m}$∈[0.6,0.8] increases progressively from time-sensitive to adaptive-resilient passengers. For familiarity-related parameters, the familiarity utility gain coefficient $\lambda_{F}$∈[0,1] and route familiarity adjustment coefficient $\xi_{L}$∈[0.5,0.7] are assigned from the upper-third interval for route-dependent passengers, with the remaining types allocated progressively lower ranges. The rationality coefficient $\mu_{m}$∈[14,16] is hierarchically initialized, with route-dependent passengers assigned to the upper interval, time-sensitive passengers to the middle interval, and adaptive-resilient passengers to the lower interval.

During parameter assignment, passenger type labels are first allocated according to predefined proportions. For each parameter, specific values are then generated through uniform random sampling within the corresponding sub-interval of the assigned passenger type. All parameters remain fixed throughout the travel process, except for the rationality coefficient $\mu_{m}$, which dynamically adjusts with waiting time and degradation level. Through the three-step mechanism of “interval partitioning – type matching – within-interval random generation,” the model establishes a heterogeneity structure that simultaneously captures categorical behavioral differences and preserves individual stochastic variability, while ensuring that all parameter values remain strictly bounded within the ranges specified in Table 4.

In the simulation, behavioral heterogeneity is implemented at the individual passenger level. For each passenger *m* entering the system, a categorical indicato$z_{m}$∈{1,2,3} is first generated. The assignment follows a discrete distribution with weights (π_1_,π_2_,π_3_), satisfying π_1_+π_2_+π_3_=1. In the baseline configuration, equal weights π_1_=π_2_=π_3_= are adopted to avoid structural bias toward any specific behavioral range.

Once $z_{m}$ is determined, the corresponding parameter sub-interval is activated for each behavioral parameter. Let the calibrated interval of a parameter be [a,b]. This interval is evenly partitioned into three sub-intervals:

[a,a+]，[a+,a+]，[a+,b].

If $z_{m}$=*k*, the parameter value for passenger *m* can be generated by uniform random sampling within the *k*-th sub-interval.

This procedure is consistently applied to all time-related, transfer-related, congestion-related, degradation-related, and familiarity-related parameters. All parameters remain fixed during the passenger’s trip except for the rationality coefficient, which dynamically evolves according to Equation (18) as a function of waiting time and degradation intensity.

Through this assignment and generation mechanism, all parameter sub-intervals can be represented within the simulated passenger population, while avoiding structural bias caused by concentrating individuals around a single representative value. The combination of probabilistic allocation and interval-based parameter generation can introduce individual-level heterogeneity while preserving consistency with the calibrated parameter ranges. This design allows the model to reflect differentiated behavioral sensitivities while maintaining numerical stability and computational tractability.

4. The route identification is based on Eq. (4) is not accurate. The formula of W_{min} is incomplete. Please check all the formulas.

**Response：**

We sincerely thank the reviewer for the valuable comment. In response to this concern, we conducted a systematic review and targeted revision of the descriptions related to the path identification procedure, the definition and usage of $W_{\min}$, and the overall consistency of the equations throughout the manuscript. The detailed revisions are described as follows.

(1) Regarding the issue that “the route identification is based on Eq. (4), which is not accurate”

In the original manuscript, the relationship between Eq. (4) and the path identification process was not expressed with sufficient precision, which may have led to the misunderstanding that the path identification procedure was directly based on Eq. (4). In fact, Eq. (4) is only used to represent the observed passenger travel information obtained from AFC data, whereas the path identification process relies on constraint conditions such as temporal consistency and transfer feasibility. To eliminate this ambiguity, the relevant description has been clarified in the revised manuscript.

(2) Regarding the definition and formulation of $W_{\min}$

We acknowledge that the original definition of $W_{\min}$ was not presented with sufficient completeness and clarity, which may have caused confusion regarding its computational meaning and role within the cost-driven search process. In the revised manuscript, we have reformulated the expression of $W_{\min}$ to explicitly define it as the minimum generalized cost among all feasible candidate paths.

In addition, the summation range has been clearly specified to indicate that the cost accumulation is restricted to links belonging to the current path, rather than all links in the network, thereby eliminating potential ambiguity.

Furthermore, we have unified the notation and logical relationships among the cost function, the pruning condition, and the definition of $W_{\min}$. The revised formulation clearly distinguishes between the cumulative cost of the current path and the global minimum cost used as a pruning benchmark. These modifications enhance the mathematical rigor, improve internal consistency, and strengthen the overall clarity of the manuscript.

**Original version：**If the cost of the current path exceeds twice the minimum cost already identified, i.e., $W(P_{q})>2\cdot W_{\text{min}}$, the extension of this path is terminated and the path is pruned[23-24]. The minimum cost is defined as follows:

$$W_{\min}=\min_{1\text{≦}\leq Q} W(P_{q})$$

The final generated path set is expressed as:

$$P=\{P_{q}\in P\text{∣}W(P_{q})\leq2\cdot W_{\min}\}$$

The set of outgoing paths $R^{(m)}$ is represented by equation (4), where $S_{o}^{(m)}$ and $S_{d}^{(m)}$ denote the entry and exit stations, respectively, and $t_{S_{o}}^{in(m)}$ and $t_{S_{d}}^{out(m)}$ represent the corresponding times of entering and exiting the stations.

$$R^{(m)}=\left(\begin{matrix} S_{o}^{(1)} & S_{d}^{(1)} & t_{S_{o}}^{in(1)} & t_{S_{d}}^{out(1)} \\ S_{o}^{(2)} & S_{d}^{(2)} & t_{S_{o}}^{in(2)} & t_{S_{d}}^{out(2)} \\ \vdots& \vdots& \vdots& \vdots\\ S_{o}^{(M)} & S_{d}^{(M)} & t_{S_{o}}^{in(M)} & t_{S_{d}}^{out(M)} \end{matrix}\right)$$

The route identification is based on Eq. (4),

**Revised version**：Since depth-first search may generate routes with significantly higher costs during path expansion, to prevent such routes from entering subsequent analysis and to control the search space, the minimum route cost is introduced as a reference to record the smallest generalized cost among the currently generated routes.At the initial stage of the search, $W_{\min}$ is not assigned a value. Once the first complete route $P_{q}$ is generated, its cost is assigned to $W_{\min}$. During subsequent route generation, $W_{\min}$ is updated according to the following rule:

$$W_{\min}=\min(W_{\min},W(P_{q}))$$

Based on the current $W_{\min}$, if the cost of a route $P_{q}$ satisfies $W(P_{q})>2W_{\min}$, the further expansion of this route is terminated. The final retained route set is expressed as:

$$P=\{P_{q}\in P\text{∣}W(P_{q})\leq2\cdot W_{\min}\}$$

After the generation of candidate routes is completed, route identification is performed within the constructed origin–destination (OD) candidate route set to determine the path that matches the passenger’s actual travel process. Automated Fare Collection (AFC) data provide information on passengers’ entry station, exit station, and corresponding timestamps, which serve as observed inputs for route identification. Accordingly, the travel attribute vector of passenger *m* is defined as:

$$\mathbf{A}_{m}=(S_{o}^{m},S_{d}^{m},t_{o}^{m},t_{d}^{m})$$

where $S_{o}^{m}$ and $S_{d}^{m}$ denote the entry and exit stations of passenger mmm, respectively, and $t_{o}^{m}$ and$t_{d}^{m}$ represent the corresponding entry and exit times. Equation (4) reflects only the passenger’s observed travel information and serves as the input for route identification, without involving route generation or evaluation. Within the generated OD candidate route set, the observed travel time information provided by Eq. (4) is used to perform temporal matching with candidate routes.

5. The original formula is ambiguous. The summation range $e_{ij}$in E may mislead readers to sum up the travel time of all links in the network, but it should actually refer to only the links included in path P_q.

**Response：**

We sincerely thank the reviewer for pointing out the ambiguity in the formulation of this equation. We acknowledge that the original representation of the summation notation was not sufficiently rigorous and may indeed have led to misunderstanding. To prevent potential confusion for readers, we have revised the equation and explicitly clarified the summation range.

**Original version：**

$$W\left(P_{q}\right)=\sum_{e_{ij}\in E} t_{e_{ij}}+\alpha\cdot t_{P_{q}}^{\text{walk}}+\beta\cdot\delta_{P_{q}}^{\text{trans}}$$

where $t_{e_{ij}}$ is the running time on segment $e_{ij}$,

**Revised version：**

$$W(P_{q})=\sum_{e_{ij}\in E:e_{ij}\subset P_{q}} t_{e_{ij}}+\alpha\cdot t_{P_{q}}^{walk}+\beta\cdot\delta_{P_{q}}^{trans}$$

where $t_{e_{ij}}$ denotes the running time of link $e_{ij}$, and $\sum_{e_{ij}\in E:e_{ij}\subset P_{q}} t_{e_{ij}}$ represents the summation of running times over the links that constitute path $P_{q}$,

6. There are also several issues with the figures. The authors should revise the figures. (1) Figure 3 does not clearly indicate the location or timing of passenger exit swiping, which is essential for understanding the path identification process.

**Response:**

We thank the reviewer for raising this important issue. We agree that the original figure did not explicitly represent the exit card-swiping step, which may have affected the clarity of the path identification logic. In the revised manuscript, we have added a “Swipe Card to Exit” state before the “Exit the Station” step to clearly indicate the exit validation process as a critical time marker. The revised Figure 3 is provided in the supplementary attachment for reference. This modification improves the logical completeness and clarity of the state transition diagram.

(2) The legend "passenger path reselection rate" appears multiple times in the same figure.

**Response：**

We appreciate the reviewer’s observation regarding the duplicated legend. In the revised version, we have removed the repeated instances of “passenger path reselection rate” and standardized the legend formatting to eliminate redundancy and enhance readability. The updated figure is included in the supplementary attachment.

Reviewer #2

1.The Bounded Rationality (BR) model relies heavily on the "rationality threshold" and weights assigned to decision dimensions (time value, transfer burden, etc.). It is unclear how these specific coefficients were determined. The authors should explicitly state whether these were calibrated using empirical data or adopted from literature. Furthermore, a sensitivity analysis on the BR threshold is necessary to demonstrate the robustness of the model.

**Response：**

We sincerely thank the reviewer for the valuable suggestion regarding the robustness of the bounded rationality (BR) thresholds. In response, we have added a dedicated sensitivity analysis subsection in the revised manuscript to systematically examine the stability of the model under varying threshold settings. The analysis focuses on the influence of threshold adjustments on path reselection behavior and related indicators.

The results indicate that within the calibrated range, model outputs vary smoothly and consistently without structural instability, suggesting that the main conclusions are not dependent on specific threshold values. When the thresholds extend beyond reasonable ranges, nonlinear deviations in model behavior emerge, further confirming the appropriateness of the calibrated settings.

**The newly added subsection is provided below:**

## 5.4 Sensitivity Analysis of Bounded Rationality Threshold Parameters

To examine the responsiveness of the proposed bounded rationality route choice model to key threshold parameters and to verify its structural stability within behaviorally plausible ranges, a systematic sensitivity analysis was conducted for the rationality coefficient ($\mu_{m}$), the waiting time tolerance threshold ($\gamma_{1}^{m}$), and the degradation tolerance threshold ($\gamma_{2}^{m}$). The analysis focuses on route reselection behavior, evaluated using the average reselection rate (R) and the choice concentration index (HHI).

(1) Sensitivity Analysis of the Rationality Coefficient ($\mu_{m}$)

The rationality coefficient $\mu_{m}$ determines the dispersion level of route choice probabilities. In this study, $\mu_{m}$ was tested over an extended interval $\mu_{m}$ ∈ [2, 40], with the calibrated range defined as $\mu_{m}$∈ [14, 16]. The numerical results corresponding to different $\mu_{m}$ values are presented in Table 8.

**Table 8. Sensitivity results for different values of** $\mu_{m}$

| $\mu_{m}$ | **Average Reselection Rate (R)** | **Choice Concentration Index (HHI)** |
| --- | --- | --- |
| 2 | 0.095 | 0.18 |
| 5 | 0.140 | 0.26 |
| 8 | 0.170 | 0.33 |
| 12 | 0.205 | 0.38 |
| 14 | 0.230 | 0.42 |
| 16 | 0.247 | 0.44 |
| 18 | 0.258 | 0.46 |
| 20 | 0.270 | 0.48 |
| 25 | 0.295 | 0.55 |
| 30 | 0.340 | 0.68 |
| 40 | 0.395 | 0.81 |

Table 8 summarizes the quantitative impact of $\mu_{m}$ on the scale of route reselection and the concentration of route choice. Based on these results, Figures 9(a) and 9(b) depict the functional relationships between $\mu_{m}$ and R, and between $\mu_{m}$ and HHI, respectively.

**Fig 9. Sensitivity analysis of the rationality coefficient (**$\mu_{m}$**)**

As shown in Table 8 and Figures 9(a)–9(b), both the average reselection rate and the choice concentration index increase monotonically with $\mu_{m}$. Within the calibrated interval $\mu_{m}$ ∈ [14, 16], the variation in R is moderate and smooth, while HHI exhibits a gradual upward trend without any structural break or abrupt transition. When $\mu_{m}$ exceeds approximately 25, the growth rate of HHI accelerates significantly, indicating that route choice becomes increasingly concentrated. Conversely, when $\mu_{m}$ is below 8, the concentration level decreases markedly, reflecting more stochastic route choice behavior. These results indicate that the model remains structurally stable within the calibrated interval, whereas extreme rationality levels may induce nonlinear amplification effects.

(2) Sensitivity Analysis of the Waiting Time Tolerance Threshold ($\gamma_{1}^{m}$)

The waiting time tolerance threshold $\gamma_{1}^{m}$ represents passengers’ tolerance for deviations from their reference waiting time. The parameter was tested over an extended interval $\gamma_{1}^{m}$ ∈ [0.2, 1.2], with the calibrated range defined as $\gamma_{1}^{m}$ ∈ [0.6, 0.8]. The corresponding reselection rates are reported in Table 8.

**Table 9. Sensitivity results for different values of** $\gamma_{1}^{m}$

| $\gamma_{1}^{m}$ | **0.2** | **0.4** | **0.6** | **0.7** | **0.8** | **0.9** | **1.0** | **1.1** | **1.2** |
| --- | --- | --- | --- | --- | --- | --- | --- | --- | --- |
| **R** | 0.430 | 0.350 | 0.265 | 0.230 | 0.198 | 0.165 | 0.120 | 0.070 | 0.035 |

Table 8 provides the numerical impact of $\gamma_{1}^{m}$ on the route reselection rate. Based on these data, Figure 10 illustrates the variation of R with respect to $\gamma_{1}^{m}$.

**Figure 10. Sensitivity analysis of route reselection behavior with respect to the waiting time tolerance (**$\gamma_{1}^{m}$**)**

As observed from Table 9 and Figure 10, the reselection rate decreases monotonically as $\gamma_{1}^{m}$ increases. Within the calibrated interval, the variation in R is smooth and continuous, with no abrupt change or non-monotonic pattern. When $\gamma_{1}^{m}$ is below 0.4, reselection behavior increases substantially; when $\gamma_{1}^{m}$ exceeds 1.0, the reselection rate declines sharply, indicating a significant weakening of the triggering mechanism. These findings suggest that the model exhibits stable responsiveness to moderate variations in $\gamma_{1}^{m}$ within the calibrated range.

(3) Sensitivity Analysis of the Degradation Tolerance Threshold ($\gamma_{2}^{m}$)

The degradation tolerance threshold $\gamma_{2}^{m}$ reflects passengers’ tolerance toward perceived service deterioration. The parameter was tested over $\gamma_{2}^{m}$ ∈ [0.1, 1.0], with the calibrated interval defined as $\gamma_{2}^{m}$ ∈ [0.4, 0.6]. The resulting reselection rates are shown in Table 10.

**Table 10. Sensitivity results for different values of** $\gamma_{2}^{m}$

| $\gamma_{2}^{m}$ | **0.1** | **0.2** | **0.3** | **0.4** | **0.5** | **0.6** | **0.7** | **0.8** | **0.9** | **1.0** |
| --- | --- | --- | --- | --- | --- | --- | --- | --- | --- | --- |
| **R** | 0.390 | 0.330 | 0.275 | 0.245 | 0.230 | 0.215 | 0.190 | 0.160 | 0.115 | 0.070 |

Table 9 presents the quantitative effect of $\gamma_{2}^{m}$ on the route reselection rate. Figure 11 further visualizes the relationship between $\gamma_{2}^{m}$ and R.

**Figure 11. Sensitivity analysis of route reselection behavior with respect to the degradation tolerance (**$\gamma_{2}^{m}$**)**

As shown in Table 10 and Figure 11, the reselection rate decreases monotonically as $\gamma_{2}^{m}$ increases. Within the calibrated interval, the variation in R remains smooth and stable. When $\gamma_{2}^{m}$ takes extreme values, the behavioral response exhibits noticeable amplification or suppression effects.

A comprehensive comparison of Tables 8–10 and Figures 9–11 indicates that, within the calibrated intervals adopted in this study, the effects of bounded rationality threshold parameters on route reselection behavior are continuous and smooth, without structural breaks or non-monotonic transitions. Although nonlinear responses emerge when parameters deviate substantially from behaviorally plausible ranges, the overall behavioral patterns remain consistent.

2. While the simulation results are logically sound, there is a lack of quantitative comparison with real-world observations.

**Response：**

We sincerely thank the reviewer for the valuable suggestion regarding the lack of comparison with real-world observational data. In response to this comment, we have incorporated actual observed data into the revised manuscript and made corresponding improvements in two aspects:

(1) In Table 6, a new column entitled “L2 (Observed)” has been added to include the actual passenger path reselection rates for comparison. In addition, the manuscript now provides a deviation range analysis among the bounded rationality model, the fully rational model, and the observed data, thereby quantitatively evaluating the differences between model outputs and real-world observations.

(2) A new Figure 6(e) has been added to present the spatiotemporal distribution of observed station passenger flow density under the L2 degraded operation scenario. Corresponding analytical discussion has also been included. Furthermore, point-by-point error statistics were conducted based on 350 station–time samples, and quantitative evaluation metrics including RMSE, MAE, mean relative error, and R² are reported. These indicators demonstrate the consistency between simulation results and observed data from both magnitude and evolutionary trend perspectives.

**The detailed revisions are provided as follows:**

①**Table 6. Peak Proportion of Passenger Path Re-selection in the Interlocking Area under Different Degraded operations**

| **Station** | **L1 (Bounded Rationality)** | **L1 (Fully Rational)** | **L2 (Actual Value)** | **L2 (Bounded Rationality)** | **L2 (Fully Rational)** | **L3 (Bounded Rationality)** | **L3 (Fully Rational)** |
| --- | --- | --- | --- | --- | --- | --- | --- |
| West Lake Cultural Square | 12.5% | 21.4% | 19.8% | 18.9% | 32.7% | 24.3% | 45.8% |
| Wulin Rd. | 15.8% | 24.6% | 24.2% | 23.6% | 36.9% | 31.6% | 50.2% |
| Fengqi Rd. | 18.3% | 27.3% | 29.4% | 28.1% | 41.5% | 38.2% | 56.4% |

Table 6 shows that as the severity of degraded operations increases, the peak path re-selection rate at stations within the interlocking area progressively rises. Specifically, the peak re-selection rate at Fengqi Rd. Station increases from 18.3% under L1 to 28.1% under L2, and further to 38.2% under L3, while Wulin Rd. Station and West Lake Cultural Square Station exhibit similar trends. This highlights the significant impact of degraded operations changes on passenger path choices, with passengers more likely to switch routes as degraded operations intensify.

To compare the consistency between model results under different rationality assumptions and actual observations, passenger route reselection rates under the fully rational and bounded rationality settings were first compared. On this basis, the observed values under the L2 degraded operation scenario were selected as the reference benchmark for further analysis. The results indicate that under all degraded operation levels, the fully rational model yields higher route reselection proportions than the bounded rationality model, and the gap between the two models widens as the degradation level increases. The difference intervals between the two model results under L1, L2, and L3 are [8.8%, 9.0%], [13.3%, 13.8%], and [18.2%, 21.5%], respectively.

Under the L2 degraded scenario, the results of both rationality assumptions were further compared with the actual observed values. At West Lake Cultural Square Station, the deviations of the bounded rationality and fully rational models are 0.9% and 12.9%, respectively; at Wulin Rd. Station, they are 0.6% and 12.7%; and at Fengqi Rd. Station, they are 1.3% and 12.1%. Correspondingly, the deviation range of the bounded rationality model is 0.6%–1.3%, whereas that of the fully rational model is 12.1%–12.9%. A clear disparity in deviation magnitude is observed between the two models, with the bounded rationality model exhibiting substantially smaller numerical deviations from the actual observations overall.

②To evaluate the discrepancy between the simulation results and actual operational data under the L2 degraded operation scenario, a point-by-point comparison was conducted using 350 “station–time” sample points derived from 14 stations and 25 time intervals within the core section affected by the degradation. The calculated performance metrics are as follows: RMSE = 0.021, MAE = 0.016, MRE = 3.8%, and coefficient of determination R^2^=0.93.Figure 6(e) presents the spatiotemporal evolution of observed station passenger flow density under the L2 degraded operation scenario. In terms of spatiotemporal distribution characteristics, the simulation results exhibit a high degree of consistency with the observed data with respect to the upward density trend at core stations, the direction of diffusion, and the timing of growth inflection points. Both display an evolutionary pattern characterized by gradual diffusion from the core area to adjacent sections, with closely aligned overall trends.

3.The simulation focuses on specific segments. In a real URT network, degraded operations on one line often trigger re-routing to parallel lines. Please clarify if the model considers network-wide path re-selection. If limited to a single-line analysis, the limitations regarding network-level spillover effects should be discussed.

**Response：**

We sincerely thank the reviewer for the valuable comment regarding whether the model accounts for network-wide path reselection and potential spillover effects. We acknowledge that the original manuscript did not sufficiently clarify the network-level scope of the simulation framework in the experimental design section, which may have led to the impression that the analysis was limited to a local segment.

In response, we have added a clarifying paragraph in Section 5.2 of the revised manuscript to explicitly state that the proposed simulation framework is constructed on the complete network topology of Hangzhou Metro Lines 1, 2, and 4. Path generation, trajectory identification, and route reselection are conducted over the full candidate path set across the entire network, allowing passengers to select alternative cross-line routes under degraded operation scenarios. The focus on the most affected interlocking section in the result presentation is intended to highlight the core propagation mechanisms triggered by degraded operations, rather than implying a single-segment modeling framework.

**The added paragraph reads as follows:**

It should be emphasized that the proposed simulation framework is established on the complete network topology of Hangzhou Metro Lines 1, 2, and 4. Path generation, trajectory identification, and route reselection are all conducted over the full candidate path set across the entire network. Consequently, under degraded operation scenarios, passengers are allowed to select alternative routes involving cross-line transfers and network-wide detours. The present study concentrates on the interlocking section with the most pronounced degradation impact for result presentation, as this area exhibits the most significant passenger accumulation and behavioral adaptation. Such a localized analytical focus is adopted to clearly demonstrate the core propagation mechanisms triggered by degraded operations, while the underlying computation and route choice processes remain network-wide.

**Minor Comments**

1.Figures 8(a) and 8(b) are quite dense. Labels for the X-axis and legends are small and difficult to read. I suggest increasing the font size and ensuring high-resolution formats.

**Response：**

We sincerely thank the reviewer for the valuable suggestion. In response to the concern that Figures 8(a) and 8(b) appear visually dense, with small x-axis labels and legend fonts that are difficult to read, we have comprehensively revised the corresponding figures. Specifically, we have increased the font sizes of the x-axis tick labels, legend text, and axis labels, and re-exported the figures to enhance overall clarity and resolution, thereby improving readability. The revised figures are provided in the supplementary attachment for reference.

2.The term "Propagation Intensity" should be explained more intuitively. What does an intensity of "0.8" represent in terms of practical operational risk?

**Response：**

We sincerely thank the reviewer for the valuable suggestion. In response, we have supplemented the practical operational interpretation of “Propagation Intensity” in the revised manuscript. Specifically, revisions were made in two locations:

(1) Following the definition of the propagation intensity metric, we added a clarification stating that this indicator reflects the transmission of accumulated passenger demand and excess queuing pressure between stations, thereby characterizing the diffusion process of congestion pressure within the network structure;

(2) In the experimental results section (corresponding to Figure 7), we added an explanation of the operational implications associated with high propagation intensity values (e.g., approaching 0.8). We clarified that such values indicate that accumulated passenger demand at core stations propagates along train operating directions and alternative routes to adjacent and downstream stations, resulting in a broader spatial overlap between rising passenger density and insufficient capacity, which may lead to regional congestion risks.

The following two additions have been made:

①Propagation intensity is used to characterize the degree to which passenger flow pressure is transmitted between stations, reflecting the diffusion capability of congestion within the network structure. When the propagation intensity approaches 0, congestion is largely confined to a single station, with limited influence on adjacent stations. When it falls within the range of 0.3–0.5, passenger flow pressure begins to spread to neighboring stations. When the value exceeds 0.7, congestion exhibits significant cross-station propagation characteristics, indicating strengthened inter-station coupling and an increased risk of regional congestion diffusion. When propagation intensity approaches 0.8 or higher, the system is typically considered to be in a high propagation risk stage, where congestion pressure at core stations is clearly transmitting to surrounding stations, potentially leading to broader operational instability.

②As shown in Fig 7, passenger flow propagation intensifies as the degraded operations de-escalates from L1 to L3. The intensity evolves from localized concentration under L1 to widespread diffusion under L3, with the affected area expanding from core stations to approximately twice the interval, accompanied by an increased propagation rate. Additionally, the propagation duration is significantly extended, shifting from rapid attenuation in L1 to sustained effects even after the peak period under L3. Overall, lower degraded operations result in stronger, and longer-lasting passenger flow propagation, following a stepwise diffusion pattern from core to adjacent and downstream stations. From a network-level perspective, the accumulated passenger demand and excess queuing pressure caused by reduced capacity in a local section propagate outward along train operating directions and passenger alternative routes. This transmission leads to simultaneous increases in inbound passenger flow and waiting passengers at adjacent and downstream stations, resulting in a broader spatial overlap between rising passenger density and insufficient capacity.

3. While generally clear, some sentences are overly long.

**Response：**

Thank you for your valuable suggestion regarding sentence structure and readability. In response to your comment, we have carefully reviewed the manuscript and revised several overly long sentences throughout the paper. During the revision process, we strictly preserved the original academic meaning and logical relationships, and only restructured sentence forms to improve clarity and readability. The specific revisions are presented below:

(1) Introduction Section

**Original sentence:**

These operational challenges collectively hinder direct observation and precise estimation of passenger flow dynamics during service disruptions, making research on human-train collaborative passenger flow estimation critically important for understanding evolving crowd movement patterns and developing effective emergency response strategies aligned with actual passenger behavior characteristics.

**Revised sentence:**

These operational challenges collectively hinder the direct observation and precise estimation of passenger flow dynamics during service disruptions. Therefore, research on human–train collaborative passenger flow estimation is critically important. Such research contributes to understanding evolving crowd movement patterns and developing effective emergency response strategies aligned with actual passenger behavior characteristics.

(2) Literature Review Section

**Original sentence:**

Although these studies have illuminated congestion phenomena under both sudden passenger flow conditions and operational disruptions from perspectives encompassing propagation rate, impact scope, and chain reactions, they have failed to effectively incorporate microscopic passenger behavior analysis.

**Revised sentence:**

Although these studies have illuminated congestion phenomena under both sudden passenger flow conditions and operational disruptions, they mainly focus on propagation rate, impact scope, and chain reactions. However, they have not effectively incorporated microscopic passenger behavior analysis.

(3) Model Contribution Section

**Original sentence:**

This framework enables dynamic simulation of train operation states and passenger flow behaviors under both normal and degraded operating conditions, thereby resolving the limitation of existing studies in adequately revealing the endogenous characteristics of passenger flow congestion propagation during service degradation scenarios.

**Revised sentence:**

This framework enables dynamic simulation of train operation states and passenger flow behaviors under both normal and degraded operating conditions. It thereby resolves the limitation of existing studies in adequately revealing the endogenous characteristics of passenger flow congestion propagation during service degradation scenarios.
